# Charting the single-cell and spatial landscape of IDH-wild-type glioblastoma with GBmap

**DOI:** 10.1093/neuonc/noaf113

**Published:** 2025-05-01

**Authors:** Cristian Ruiz-Moreno, Sergio Marco Salas, Erik Samuelsson, Mariia Minaeva, Ignacio Ibarra, Marco Grillo, Sebastian Brandner, Ananya Roy, Karin Forsberg-Nilsson, Mariette E G Kranendonk, Fabian J Theis, Mats Nilsson, Hendrik G Stunnenberg

**Affiliations:** Department of Molecular Biology, Faculty of Science, Radboud University, Nijmegen, The Netherlands; Prinses Máxima Centrum for Pediatric Oncology, Utrecht, The Netherlands; Science for Life Laboratory, Department of Biochemistry and Biophysics, Stockholm University, Stockholm, Sweden; Science for Life Laboratory, Department of Biochemistry and Biophysics, Stockholm University, Stockholm, Sweden; Institute of Computational Biology, Helmholtz Zentrum München, German Research Center for Environmental Health, Neuherberg, Germany; Institute of Computational Biology, Helmholtz Zentrum München, German Research Center for Environmental Health, Neuherberg, Germany; Science for Life Laboratory, Department of Biochemistry and Biophysics, Stockholm University, Stockholm, Sweden; Division of Neuropathology and Department of Neurodegenerative Disease, UCL Queen Square Institute of Neurology, Queen Square, UK; Department of Immunology, Genetics and Pathology, Science for Life Laboratory; Uppsala University, Uppsala, Sweden; Department of Immunology, Genetics and Pathology, Science for Life Laboratory; Uppsala University, Uppsala, Sweden; Prinses Máxima Centrum for Pediatric Oncology, Utrecht, The Netherlands; Institute of Computational Biology, Helmholtz Zentrum München, German Research Center for Environmental Health, Neuherberg, Germany; Science for Life Laboratory, Department of Biochemistry and Biophysics, Stockholm University, Stockholm, Sweden; Department of Molecular Biology, Faculty of Science, Radboud University, Nijmegen, The Netherlands; Prinses Máxima Centrum for Pediatric Oncology, Utrecht, The Netherlands

**Keywords:** glioblastoma, hypoxia, spatial transcriptomics, single-cell atlas, tumor organization

## Abstract

**Background:**

Glioblastoma (GB), particularly IDH-wild type, is the most aggressive brain malignancy with a dismal prognosis. Despite advances in molecular profiling, the complexity of its tumor microenvironment and spatial organization remains poorly understood. This study aimed to create a comprehensive single-cell and spatial atlas of GB to unravel its cellular heterogeneity, spatial architecture, and clinical relevance.

**Methods:**

We integrated single-cell RNA sequencing data from 26 datasets, encompassing over 1.1 million cells from 240 patients, to construct GBmap, a harmonized single-cell atlas. High-resolution spatial transcriptomics was employed to map the spatial organization of GB tissues. We developed the Tumor Structure Score (TSS) to quantify tumor organization and correlated it with patient outcomes.

**Results:**

We showcase the applications of GBmap for reference mapping, transfer learning, and biological discoveries. GBmap revealed extensive cellular heterogeneity, identifying rare populations such as tumor-associated neutrophils and homeostatic microglia. Spatial analysis uncovered 7 distinct tumor niches, with hypoxia-dependent niches strongly associated with poor prognosis. The TSS demonstrated that highly organized tumors, characterized by well-defined vasculature and hypoxic niches, correlated with worse survival outcomes.

**Conclusions:**

This study provides a comprehensive resource for understanding glioblastoma heterogeneity and spatial organization. GBmap and the TSS provide an integrative view of tumor architecture in GB, highlighting hypoxia-driven niches that may represent avenues for further investigation. Our resource can facilitate exploratory analyses and hypothesis generation to better understand disease progression.

## Graphical Abstract

**Graphical Abstract:**
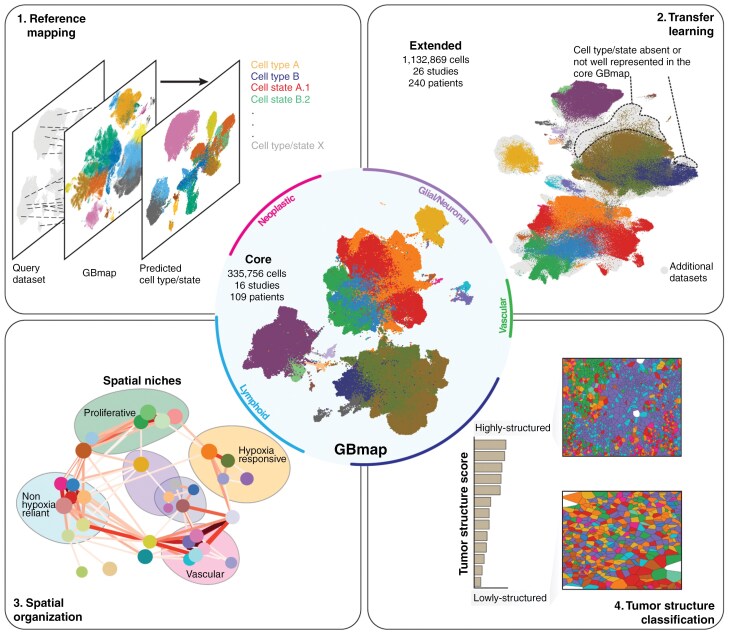

**Key Points**

▪ GBmap integrates 1.1 M single-cell transcriptomes, revealing glioblastoma heterogeneity.
▪ Spatial analysis identifies hypoxia-driven niches linked to poor prognosis.
▪ TSS quantifies tumor organization, offering new prognostic insights.

**Importance of the Study**

This study presents GBmap, a comprehensive single-cell and spatial atlas of IDH-wild-type glioblastoma, integrating over 1.1 million cells from 240 patients. GBmap enables robust cell-type annotation, transfer learning, and discovery of rare populations like tumor-associated neutrophils. Using high-resolution spatial transcriptomics, we identified 7 distinct tumor niches, revealing hypoxia-dependent niches as key drivers of tumor progression. We introduced the Tumor Structure Score, linking highly organized tumors with poor prognosis. These findings refine our understanding of glioblastoma heterogeneity, spatial organization, and their impact on patient outcomes, highlighting directions for future research and potentially informing prognostic stratification. GBmap serves as a foundational resource for future glioblastoma research, providing insights into the tumor microenvironment and its role in disease progression.

GB is the most common malignant brain neoplasm in adults and continues to have a poor prognosis despite developments in multimodal therapy.^1^ Recent advances in the molecular profiling of individual cells have unraveled a remarkable cell heterogeneity of the neoplastic cells and the tumor microenvironment (TME), especially the immune compartment.^2–4^

To better capture the molecular underpinnings of transcriptomic variation, it is necessary to gather and integrate large cohorts that can empower understanding of the disease mechanisms and capture the phenotypic features of GB. Single-cell RNA sequencing (scRNA-seq) has been instrumental in characterizing the tumor cellular makeup, and its adoption to study gliomas has led to the generation of a plethora of datasets that vary in size, sequencing protocols, cell enrichment, and regional sampling, amongst others.^5–10^ Successful harmonization of such diverse datasets can help chart the TME with higher precision. Integrating scRNA-seq data has been one of the main challenges in the field, and reducing the technical variance is paramount in generating a high-quality reference map.^11–15^

A significant limitation of traditional gene expression studies is the loss of spatial context due to tissue dissociation. Spatially resolved transcriptomics (ST) allow for transcriptomic profiling while preserving the spatial architecture of tissues, providing crucial insights into the organization of cells within the tumor niches. This technology has begun to reveal the spatial heterogeneity of GB,^16–18^ offering new perspectives on its pathobiology.

We set out to build the ‘GBmap’ (Fig 1a). At its core, it integrates multiple scRNA-seq datasets, providing a harmonized annotation at different granularity levels. We demonstrate the applicability of the GBmap to annotate and integrate newly generated data robustly. Through transfer learning, we gathered more than 1.1 million cells, which led to the identification of under-represented cell (sub)types, such as neutrophils or homeostatic microglia. Finally, the GBmap assisted in finely mapping cell states in high-resolution ST data to chart the architecture in GB.

**Figure 1.**
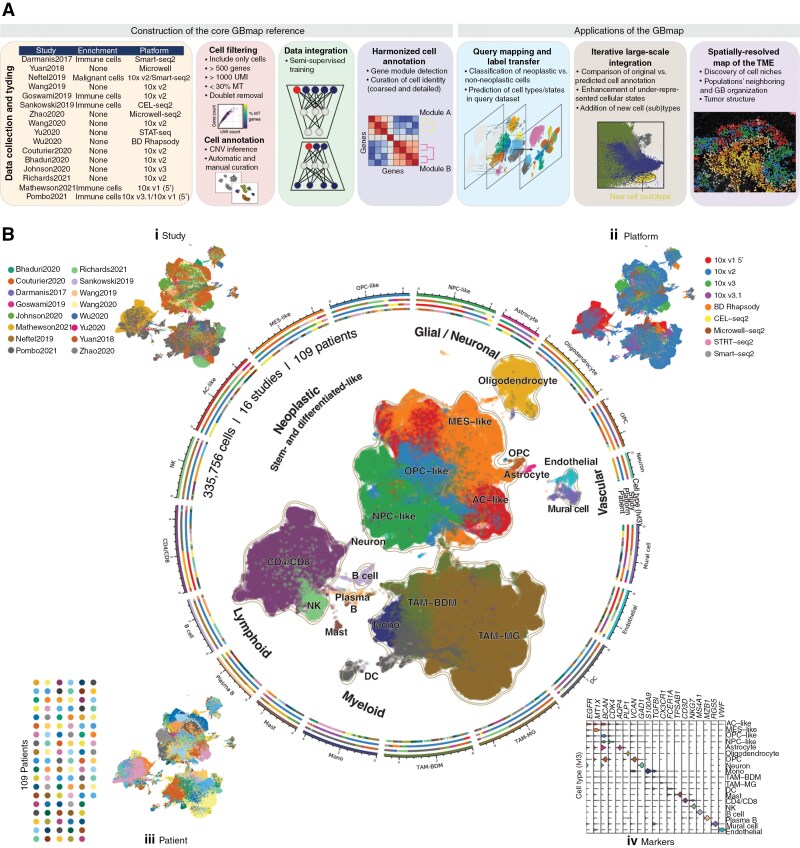
Construction of the core GBmap reference. (A) Study design and computational analysis summary. It shows the main steps taken to build and annotate the core GBmap reference and its different applications. (B) UMAP (uniform manifold approximation and projection) of the GB atlas (core GBmap) over ~330 000 cells (16 studies and 109 patients) after data integration and batch correction using SCANVI. The corner insets and colored radial tracks depict (i) the study, (ii) the sequencing platform, (iii) the patient, and (iv) marker-gene expression. The axis outside the circular plot shows the log scale of the total cell number for each cell type (level-3 annotation).

## Materials and Methods

### Tissue Collection and RNA Sequencing

Fresh GB tissue sections were obtained from primary resections and biobank resources under ethical approval. For single-nuclei RNA sequencing, cryosections were processed to isolate nuclei using mechanical homogenization and fluorescence-activated sorting.

### Single-Nuclei RNA Sequencing De Novo GB Specimens

Isolated nuclei were counted and loaded onto a 10× Genomics Chromium instrument (3ʹ v3 chemistry). Libraries were prepared according to the manufacturer’s protocol, followed by the quality assessment on a Bioanalyzer. Sequencing was carried out on an Illumina NovaSeq. Raw data were processed with Cell Ranger, generating gene count matrices for downstream analysis.

### Data Integration and GBmap Construction

Public scRNA-seq datasets (from 16 studies) were compiled and quality filtered (≥500 genes, ≥1000 UMIs, <30% mitochondrial reads). To integrate these heterogeneous datasets, we benchmarked multiple batch correction tools using a standardized scIB pipeline. scANVI within the scArches transfer-learning framework was selected for its optimal balance between technical variance removal and biological signal preservation. Malignant vs. nonmalignant status was inferred by copy number variation (iCNV) profiling (SCEVAN), leveraging known diploid reference cells. To refine annotation granularity, each major cell lineage (eg, neoplastic, myeloid, vascular) was further characterized via Hotspot, which groups co-expressed genes into modules. The resulting GBmap atlas initially comprised over 330 000 cells, later extended to over 1.1 million cells, with robust and hierarchical cell-type annotation via clustering, module detection, and differential gene expression analyses.

### Reference Mapping Using GBmap

The Azimuth and scArches algorithms were applied to identify shared anchor features between the query and the core GBmap to map external datasets onto the GBmap reference. In addition, a leave-one-out cross-validation strategy was employed: individual datasets were sequentially withheld from the reference and then remapped using the remaining data. This procedure assessed the consistency and robustness of cell-type label transfer. Additionally, normal human cortex datasets were mapped to confirm the robust separation of neoplastic and nonneoplastic cells.

### Spatial Transcriptomics and Cell Typing

A targeted RNA in situ sequencing (RNA-ISS) approach was performed on 13 GB samples. A custom gene panel (209 genes) was designed based on the GBmap signatures to accurately capture malignant and nonmalignant cell states. Tissue sections underwent fixation, permeabilization, hybridization with padlock probes, ligation, and amplification. Fluorescence imaging and subsequent decoding using custom pipelines enabled probabilistic cell typing (pciSeq), mapping individual cells to their transcriptomic identities in situ.

### Tumor Structure Scoring

Spatial organization was quantified by performing neighborhood enrichment analysis (via Squidpy) on decoded cell maps. These enrichment scores were aggregated into a Tumor Structure Score (TSS), which reflects the degree of tissue organization. The TSS was correlated with vasculature characteristics and patient outcomes.

### Survival Analysis

Gene expression and clinical metadata for TCGA-GBM were retrieved via TCGAbiolinks. Patients were stratified by the expression of TSS-associated genes, and survival was evaluated using Kaplan–Meier curves and a Cox proportional hazards model. Age, sex, and MGMT status were included as covariates. Statistical procedures were implemented in R. Further details are provided in Supplementary Material.

## Results

### Building the Single-Cell GB Core Reference

To establish a GB core reference, we gathered and selected only scRNA-seq profiles of IDH-wildtype tumors from 16 studies across multiple platforms and diverse sample preparation strategies, obtaining over 330 000 cells from 109 patients (Figure 1A; **Table S1**). We included all clinicopathological information when available (**Figure S1A-B**). To select the best integration strategy, we scored different batch correction algorithms using the scIB pipeline.^11^ We concluded that scANVI^19^ provided the best balance in reducing the technical variance and preserving biological information (Figure 1B i-iii, **Figure S2A-C**, Methods). Next, we measured cell-type predictions and cross-prediction consistency within the core reference to assess the potential limitations of data integration. By the leave-one-out approach across datasets and technologies (**Figure S2D**), we evaluated the consistency of cell-type labels and biological variability across multiple datasets and potential batch and technology-specific biases. Our systematic analysis revealed that cell-type predictions within the core reference are robust and reproducible across datasets, maintaining high consistency in most combinations between cell types and datasets, as well as across different technologies (**Figure S2E and F**). Despite overall prediction agreements, prediction accuracies were lower in some instances. Specifically, in the Bhaduri2020, Yu2020, and Yuan2018 datasets, there was confusion among different cancer states. These cases of sub-celltype “misclassifications” might be expected since cancer cells may exist in so-called “hybrid states,” and differentiating between them may also be a difficult task since they lack one predominant identity.^7^ Thus, our results highlight the impact of dataset composition on queried dataset predictions and underscore the necessity of creating a comprehensive reference to ensure a thorough characterization of the cancer cell subtypes and robust cell signatures.

### Cell-Type Annotation and Label Harmonization

Following integration, we harmonized and re-annotated the cells in a hierarchical manner. Initially, we divided the cells into neoplastic, recognized by the overall presence of iCNV, and nonneoplastic (neuronal/glial, myeloid, lymphoid, and vascular; **Figure S2G**). Next, we conducted cell identity classification at a low granularity level (level-2 and -3 annotation). Neoplastic cells (38%) globally converged into 2 central cellular phenotypes: stem/progenitor- and differentiated-like cancer cells. Nonneoplastic cells included: (a) the innate immune compartment (39%) consisted of dendritic cells and tumor-associated macrophages, the latter further divided into blood-derived monocytes/macrophages (BDM) and resident microglia (MG); (b) tumor-infiltrating lymphocytes (17%), mainly constituted of T CD4+/CD8 + and NK, and to a lesser extent, B/plasma and mast cells; (c) nonneoplastic neuronal/glial (5%) and (d) vascular cells (1%; Figure 1B**, center and iv**). Due to the varying study designs and enrichment strategies across the datasets included in the GBmap, not all cell types are equally represented in every sample, and the proportions of cell types may not accurately reflect the reality of the tumor.

**Figure 2.**
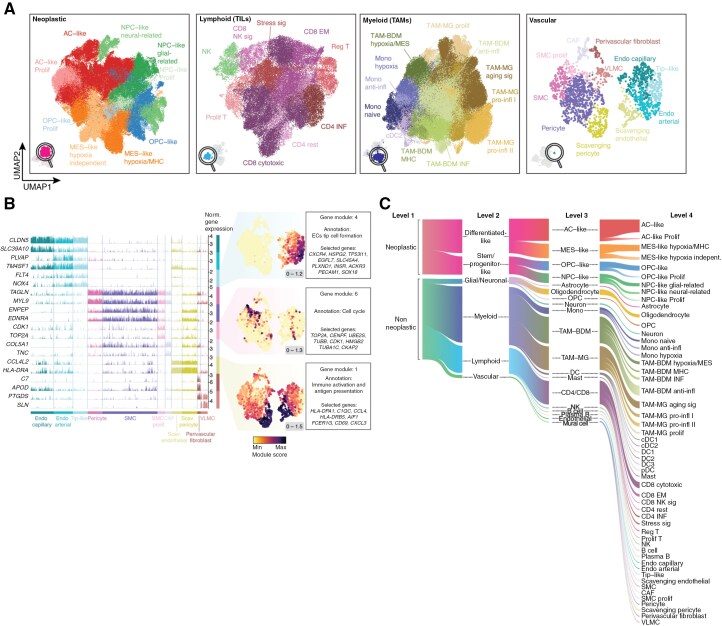
Categorization of cell states in the GBmap. (A) UMAP and cell annotation of sub-clustered neoplastic, myeloid, lymphoid, and vascular territories (level-4 annotation, detailed cell states). Harmonized cell phenotypes were obtained after identifying robust gene modules using Hotspot. (B) Expression of representative genes (left) of the different cell (sub)types part of the tumor vasculature. Each bar represents an individual cell, and the height shows the magnitude of the gene expression. On the right-hand side, enrichment scores of selected gene modules show signatures associated with tip-like cell formation (module 4), cell cycle (module 6), and immune activation (module 1). The minimum and maximum module scores are shown in the bottom right of each panel. (C) A comprehensive depiction of the cellular composition within GBmap, ranging from broader classifications (level 1) to more intricate distinctions (level 4) through a harmonized cell annotation scheme. Within the hierarchy of cell types, the initial level (1) encompasses the most general annotations (neoplastic vs. nonneoplastic). Subsequent higher levels (2–4) progressively deconstruct these broader labels into more nuanced categorizations.

Subsequently, we defined representative phenotypes for each category using gene modules (level-4; **Table S3**). Neoplastic cells displayed consistent modules associated with cellular states that mimic astrocyte (AC)-like, neural precursor cell (NPC)-like, oligodendrocyte percussor cell (OPC)-like, and mesenchymal (MES)-states as defined by Neftel et al.^7^ (Figure 2A**- Neoplastic and****Figure S3A,B****).** Additional (sub)programs within the cancer phenotypes included hypoxia and MHC-II/cytokine modules, enriched in MES-like cells, and enhanced cell proliferation in OPC/NPC- and AC-like (**Figure S3C**).

Within the immune compartment, TAM-BDM displayed up-regulation of gene programs associated with classical monocytes, interferon-induced genes, pro-inflammatory cytokines, and tumor-supportive chemokines.^20^ A subset was characterized by MES-like genes (*VIM, CSTB*), matching the MES-like myeloid phenotype.^21^ Additional gene modules, such as a hypoxia-responsive program, were also enriched in the MES-like BDM subtype (Figure 2A**and****Figure S3D**). TAM-MG exhibited up-regulation of pro-inflammatory genes, interferon response, immune activation program, and an aging-like transcriptional pattern with high expression of *SPP1*, *APOE/C,* and *BIN1*^22^ (Figure 2A**and****Figure S3D**). CD4 + T cells fell into the category of regulatory, INF signature, and effector memory cells in a polarization state^23^ (Figure 2A**and****Figure S3E**). CD8 + T cells expressed high levels of genes associated with cytotoxicity, effector memory-associated programs, and cell proliferation. In addition, we detected stress and CD8 + NK-associated signatures, which were recently validated in GB^24^ (**Figure S3E**).

In the tumor vasculature, we characterized the cells that are part of the GB blood vessels, including endothelial and mural cells. Endothelial cells (ECs) could be further classified into tip-, capillary- and arterial-like cells^25^ (Figure 2B). Arterial-like ECs showed upregulation of *PLVAP*, a marker typically associated with fenestrated morphology in high-permeable capillaries and venous vessels, suggesting a novel subtype in GB (Figure 2B). Mural cells included pericytes, fibroblasts, smooth muscle, and vascular leptomeningeal cells (Figure 2A**and****Figure S3F**). We were able to chart a subpopulation of cells that expressed marker genes associated with cancer-associated fibroblasts (CAFs), a recently recognized cell subtype in GB.^26,27^ CAFs were related to the upregulation of vascular membrane remodeling and pro-angiogenic genes (*TNC*)^28^ (Figure 2B). Notably, an unconventional phenotype featured by immune activation and antigen presentation was identified in a subcluster of ECs and pericytes (Figure 2B). The presence of this immune-enriched signature was recently discovered in GB ECs.^29^ Finding these signatures in pericytes was facilitated by our large-scale data integration, which allowed us to gather a significant number of cells and overcome the technical difficulties that may have limited previous studies in enriching vascular cells. These so-called scavenging cells might be induced by tumor-secreted cytokines, as suggested in other tumor types.^30^

We validated the consistency of the harmonized cell states in our core reference by creating a version using only the most common platform (10X), which replicated the cell (sub)types gene expression patterns, supporting the robustness of our integration and label (**Figure S4C**). Finally, we calculated Shannon’s Entropy ensuring a broad donor representation within most clusters (lowest score of 0.58, median of 49 donors per cluster, range of 3–99; **Figure S4A,B**). However, certain cell subtypes, notably DC subtypes, exhibited lower entropy scores, reflecting a more restricted donor distribution. These dendritic cells predominantly originated from the Pombo-Antunes et al. study, where CD45 + selection was employed to enrich this rare population within the GB TME, where they represent less than 1% of immune cells.^31^ We hypothesize that their low entropy scores may stem from this enrichment approach and the limited prevalence across patients. Nonetheless, potential residual batch effects cannot be entirely ruled out as a contributing factor to the observed entropy variability.

In summary, we created GBmap, a robust and diverse GB core reference that comprehensively delineates cell types and states from coarse to refined cell identity (Figure S2C**and****Table S4**).

### Reference Mapping and Cell Sub(type) Predictions Using GBmap

**Figure 3.**
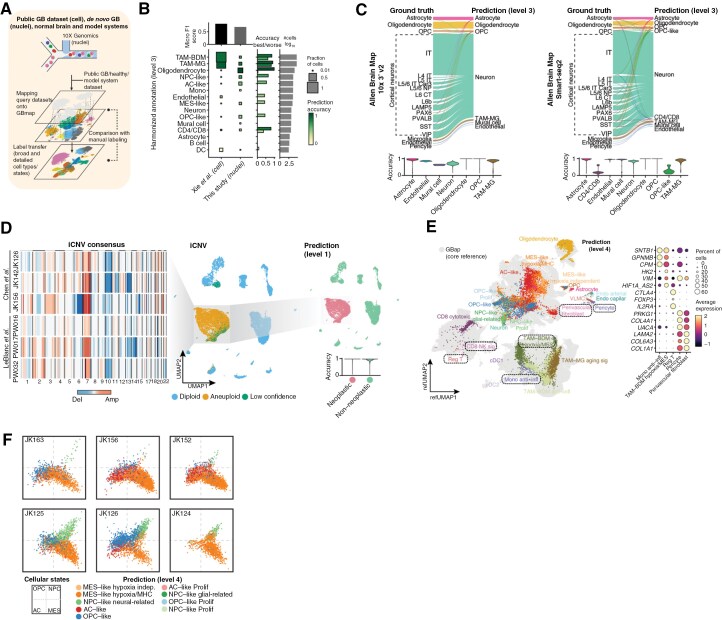
Reference mapping using GBmap. (A) Schematic outlines the experimental and in silico pipeline. (B) The square plot highlights an accuracy value retrieved from a query-to-reference mapping task of labeled datasets (x-axis) when queried onto the core GBmap. The y-axis indicates the cell-type classification performance within datasets (rows normalized). The top barplot indicates the overall classification performance of the column dataset, adjusted by cell-type numbers. The right bar plots indicate the best and worst accuracies per cell type (greens) and the total number of cells. (C) Sankey plot compares the original cell labeling of human cortex brain cells from the Allen Brain Map initiative onto the core GBmap (predicted cell types annotation level 3). Bottom violin plots display the prediction scores for each dataset. (D) UMAP of the in silico mix of human cortex brain cells from the Allen Brain Map initiative and 6 GB samples from published studies, colored by iCNV. The left heatmap shows the iCNV consensus for each GB sample. UMAP on the right depicts the in silico mix colored by predicted cell type (annotation level 1). The bottom, violin plot shows the prediction scores. (E) Projection and cell-type prediction of detailed cell states (level-4 annotation) of de novo mapped query cells (this study) onto the GBmap UMAP. On the right is the expression of gene markers from selected cell states (dotted lines in the UMAP projection). (F) Two-dimensional representation of cellular states (cancer cells) from selected patient-derived explants, colored by GBmap-predicted cell type (annotation level 4). It shows an overall agreement between the malignant GB signatures defined by Neftel et al. and the GBmap labels.

With harmonized cell annotation at hand, we explored the GBmap’s ability to assess external generalization performance and recognize cell (sub)types in new datasets. In addition, we tested the prediction capabilities when the query data was generated using nuclei instead of cells. First, we used 2 datasets external to the GBmap: (1) Xie et al.^29^ dataset (cell) and (2) our de novo (nuclei) data (Figure 3A**and****Table S5**). The cell-type annotation of these external datasets was done by expert-based cell-type annotation, following the same approach as GBmap but without using it. The cell-type label transfer micro F1 scores indicate robust performance for the Xie et al. dataset (0.81) and a moderate level of performance for our de novo dataset (0.69; Figure 3B). When assessing predictions, the external remapping reanalysis also highlighted phenotypes with lower transfer-label accuracies, such as cancer states and tumor-associated macrophages. These cases of sub-cell type “misclassifications” might be expected. For instance, differentiating between TAM-MG and TAM-BDM can be a difficult task as they may acquire similar markers and functional states under the influence of the GB TME, leading to a significant overlap in their identities.^32^

Our analysis indicates that GBmap could predict nonneoplastic and neoplastic cells, supported by the presence of iCNV in the predicted malignant cells (**Figure S5A,B**). To evaluate the accuracy and robustness of the GBmap signatures, we conducted complementary analyses to challenge the ability of the reference mapping to differentiate nonneoplastic from neoplastic cells and generate predictions across different sequencing platforms. We mapped 2 normal human cortex datasets (Smart-seq2 and 10× v2) from the Allen Brain Map initiative. Our analysis revealed that cells matched their original cell identity. Specifically for the 10x dataset, we observed no misclassification of the main predicted populations (annotation level 3). For the Smart-seq2 cohort, we found that less than 1% of cells (63/49,417) were misassigned with low accuracy scores, suggesting mislabeling or technology biases (Figure 3C**and****Figure S5C**). We additionally challenged the reference mapping by creating an *in silico* mix of the Allen Brain Map with GB donors profiled with 2 other platforms (Chen et al.^33^ – Microwell-seq and LeBlanc et al.^*34*^—10× v3; **Figure S5D**). We identified iCNV beforehand in the GB datasets and classified the cells as either aneuploid or diploid (Figure 3D). Our results showed that the GBmap could robustly predict nonneoplastic and neoplastic cells with accuracy scores above 90% (annotation level 1; Figure 3D).

The GBmap predictions can extend to the finest cell subtypes. For instance, we could identify TIL subsets in our 11 GB donors with an expression pattern of regulatory T cells, typified by the high expression of *CTLA4, IL2RA,* and *IKZF2,* or CD8 + NK signature cells featuring up-regulation of *KLRC1* and *GNLY* (Figure 3E). These populations were not detected without the label transfer provided by the GBmap, not even by sub-setting and re-clustering (**Figure S5E**). Likewise, other cell subtypes that could not be confidently classified in the standalone analysis were recognized as anti-inflammatory monocytes, hypoxia/MES-like TAM-BDM, and perivascular fibroblasts (Figure S3E). Finally, we extended the application of the GBmap-based reference mapping to patient model systems and assessed predictions when strong batch effects were present. To illustrate this, we obtained GB patient-derived explants data.^34^ Despite the fact that each PDE sample clustered independently, using the GBmap effectively assigned predictions for each cancer cell while discriminating normal populations (Figure 3F**and****Figure S5F**).

Taken together, the GBmap facilitated robust predictions of the cellular composition in queried data.

### Large-Scale Integration by Transfer Learning Using GBmap

**Figure 4.**
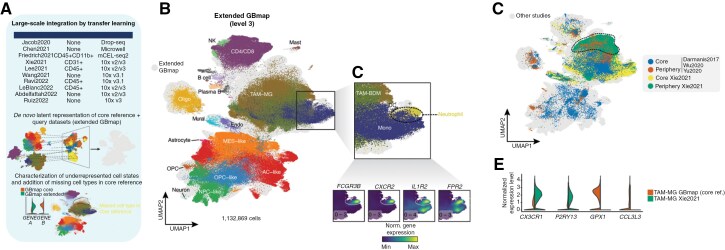
Transfer-learning capabilities of the scArches GBmap model. (A) Diagram of comparison and machine-learning process applied to public and our own dataset. (B) Updated UMAP of the integrated GBmap and query datasets (extended GBmap). Cells from the integrated studies are colored gray using the neural network model developed in scArches. Cells from the GBmap core reference are colored by level-3 annotation. (C) Close-up of UMAP region identifying the neutrophil cluster, which was not present in the core GBmap. Below is the expression of classical neutrophil gene markers. The minimum and maximum normalized gene expression values are shown in the bottom left of each panel. (D) Projection of multi-sector biopsy datasets onto the extended GBmap, colored by region (core or periphery). (E) Expression of selected phenotypical microglia (CX3CR1, P2RY13) and tumor-induced genes (GPX1, CCL3L3) contrasting TAM-MG from the core GBmap and the predicted microglial cells on the Xie2021 dataset.

While using a predetermined reference map provides rapid interpretation of new datasets, it can also constrain the discovery of novel cell phenotypes that were not captured in the original reference. scArches-SCANVI addresses this by updating and expanding the trained model, thus avoiding the limitation of strictly adhering to the reference map. To exemplify this, we ‘upgraded’ the core GBmap with new datasets that became available after constructing the trained model (Figure 4A**and****Table S1**). The outcome yielded a joint embedding resulting in the extended GBmap (>1.1 million cells; Figure 4B). Our model recapitulated the major cell groups (level-3) when comparing the label transfer (predicted cell type) to the newly mapped datasets (original annotation) enabling consensual annotation (**Figure S6A**). Of note, following large-scale integration, we detected a cluster with a high expression of neutrophil markers (*FCGR3B, CXCR2, FPR2*; Figure 4C). Importantly, tumor-associated neutrophils were absent in the core GBmap reference likely for technical/procedural reasons and could now be retrieved in the queried datasets.

Furthermore, by examining TAM-MG cells in both the core and the extended GBmap (Figure 4D), we identified a subset expressing higher levels of *bona fide* markers of a homeostatic microglial signature (*CX3CR1*, *P2RY13*) and lower expression of catabolic (*GPX1*) or cell activation (*CCL3L3*) processes (Figure 4E). These cells mainly belonged to the integrated Xie2021 dataset (Figure 4D). The multi-sector biopsy performed by Xie et al. showed that the histological assessment of the neighboring tumor tissue largely concurred with normal brain microanatomy. Analysis of the extended GBmap hinted towards the enrichment of homeostatic MG, which was in line with the region (peripheral area) from which the cells were taken. Due to the higher number of cells profiled by Xie et al. compared to previous multi-sector biopsy studies included in the core reference^35–37^ (**Figure S6B**), the divergence between “naïve” resident MG and TAM-MG was drawn out during the transfer-learning process.

These results provide a framework for GBmap to “learn” when new data is added and enable the discovery of (disease) specific cell states.

### Spatial Organization and Niche Identification in GB

**Figure 5.**
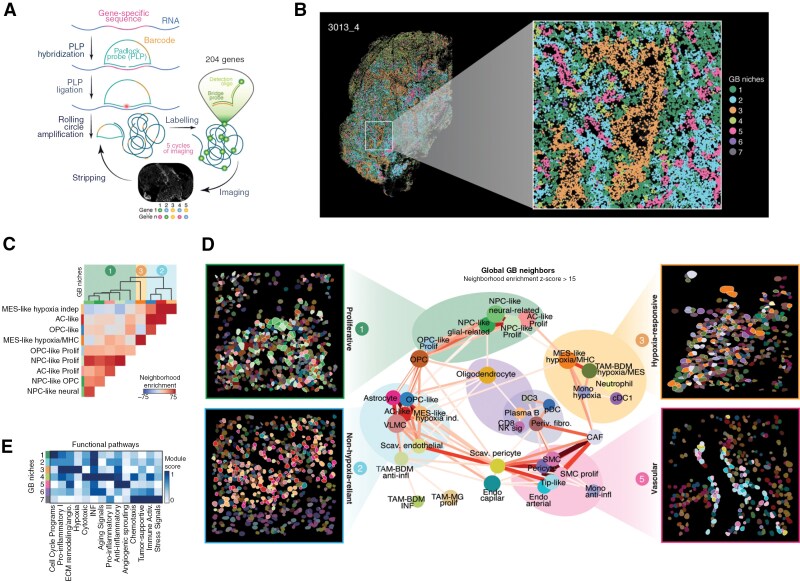
Reconstruction of the GB architecture at single-cell resolution. (A) Schematic representation of the experimental RNA-ISS workflow. (B) Representative GB section showing the identified niches. The niches identified are represented by coloring individual cells (nodes) by the niche they have been assigned to. Edges connect each cell with their closest 15 cells, used to define the composition of their niche. (C) Neighborhood enrichment scores, represented as a heatmap, between the different neoplastic populations. (D) Graph representing the neighborhood enrichment between different cell types in space, considering a radius of 40 μm. Cell types are represented as nodes, and edges connecting cell types are shown for pairs of cell subtypes with a neighborhood enrichment z-score above 15. The edge’s color and width represent the specific neighborhood enrichment z-score, ranging from 15 (thinnest) to 70 (thickest). Colored polygons on the graph’s back aim to outline the main niches. For the most abundant niches (proliferative, hypoxia-responsive, non-hypoxia-reliant, and vascular), cell type maps of specific regions of interest (selected ROIs) representing each niche are included. Segmented nuclei are colored according to the most probable predicted cell subtype according to pciSeq, using RNAISS data. A white contour line surrounds cell types enriched on each of the niches. (E) Heatmap representing the scores of GB modules correlated with the functional annotation given to each niche.

Leveraging the detailed cellular annotations derived from GBmap, we employed a high-resolution ST platform (RNA-ISS^38^) to investigate how cell states are organized. We aimed to spatially contextualize the functional heterogeneity identified in GBmap, linking specific niches with the tumor’s structural complexity. GBmap gene signatures were used as the foundation to create a customized gene panel that can identify GB’s cell (sub)types. We generated spatial gene maps using targeted probes within intact tissue at cellular resolution (Figure 5A). Thirteen distinct GB specimens were analyzed, charting the distribution of 988 901 cells (interactive viewer: https://gbmap-iss.serve.scilifelab.se). For each sample, we performed cell segmentation and assessed cell-type identity by employing probabilistic cell typing by in situ sequencing (pciSeq)^39^ (**Figure S7A**, Methods). To evaluate the ability of the panel to identify cell states confidently, we generated a confusion matrix for the pciSeq-derived cell state scores for all samples for annotation levels 3 and 4 in the GBmap (**Figure S7B–C**). Overall, the cell state assignment is confident, with the main areas of confusion found between NK and T cells (CD4 + and CD8+), as well as endothelial and mural cells for annotation level 3, TAM-BDM/-MG and NPC-/OPC-like subtypes, and T cell subtypes for annotation level 4 (**Figure S7B**). Although some cell-type assignments may have lower probabilities due to inherent challenges in distinguishing closely related cell states, the robustness of the spatial patterns across the dataset supports their inclusion in the analysis. This approach ensures that the overall spatial architecture and niche-specific features remain accurately represented.

We devised a 2-pronged analytical strategy for using the generated cell maps to reveal recurring cell-type distribution patterns. First, we set out to identify recurrent cellular niches. To achieve this, we defined the cellular microenvironment of each cell by assessing the cell-type identity of the neighbors within a 40 µm radius of each cell. Second, we identified cell-type pairs with a high mutual enrichment by considering a cell-centric evaluation of the cell neighbors and neighborhood enrichment analysis. Through clustering and neighborhood refining, we consistently identified 7 niches across samples (**Figure 5B. and****Figure S7D**).

The organization of each malignant phenotype could be summarized in 3 neoplastic-enriched niches. Niche 2 (light blue) stands out with a strong neighborhood enrichment between MES-like-hypoxia-independent and glial-like (AC/OPC-like) cancer cells colocalized in the same regions (Figure 5C). Other nonneoplastic cells, such as astrocytes and vascular cells, were also found in the vicinity (Figure 5D**bottom left, and****Figure S7E**). Niche 3 (orange) was dominated by MES-like-hypoxia/MHC cells, showing a high self-enrichment score and low enrichment with other neoplastic phenotypes indicating they occupy distinct locations within the tumors (Figure 5C). This niche contained different hypoxia-associated cell types, including mono/TAM-BDM and neutrophils (Figure 5D**top right, and****Figure S7E**). Functional signatures show that niche 3 was the only tumor compartment strongly associated with the up-regulation of genes involved in hypoxia response (Figure 5E). Lastly, neoplastic signatures such as NPC-like and cancer-proliferating phenotypes were found to colocalize in niche 1 (dark green), enriched with cell cycle-related genes (Figure 5C–E**, top left**). Self-enrichment scores for these populations were lower than for other neoplastic phenotypes, suggesting that these populations are less likely to form extensive compartmentalized structures. Based on their cellular composition and functional signature enrichments, we named these tumor-dominant niches as “proliferative” (niche 1), “non-hypoxia-reliant” (niche 2), and ‘hypoxia-responsive’ (niche 3; Figure 5D).

Additionally, we identified 4 niches dominated by non-cancerous cells (niches 4-7). Niche 5 comprised the vasculature (‘vascular’ niche—pink; Figure 5D**, bottom right**). The predominant presence of oligodendrocytes, neurons, and TAM-MG characterized niche 6 (purple). This niche also exhibited frequent co-localization with NPC-like cancer cells (**Figure S7E**). Anatomically, this compartment was located mainly in the tumor periphery and labeled as ‘peripheral’ niche (**Figure S7F**). Its cell composition aligns with recent studies that highlighted the presence of NPC-like cells in the GB infiltrative margin.^40,41^ Niche 4 (light green) was primarily associated with myeloid cells diffusely distributed within the tumor (**Figure S7E,F**). We referred to these structures as “immune interwoven.” Finally, we identified a ‘perivascular’ compartment (niche 7) characterized by perivascular fibroblasts, pDCs, and plasma cells (**Figure S7E,F**).

### Tumor Structure Classification in GB

**Figure 6.**
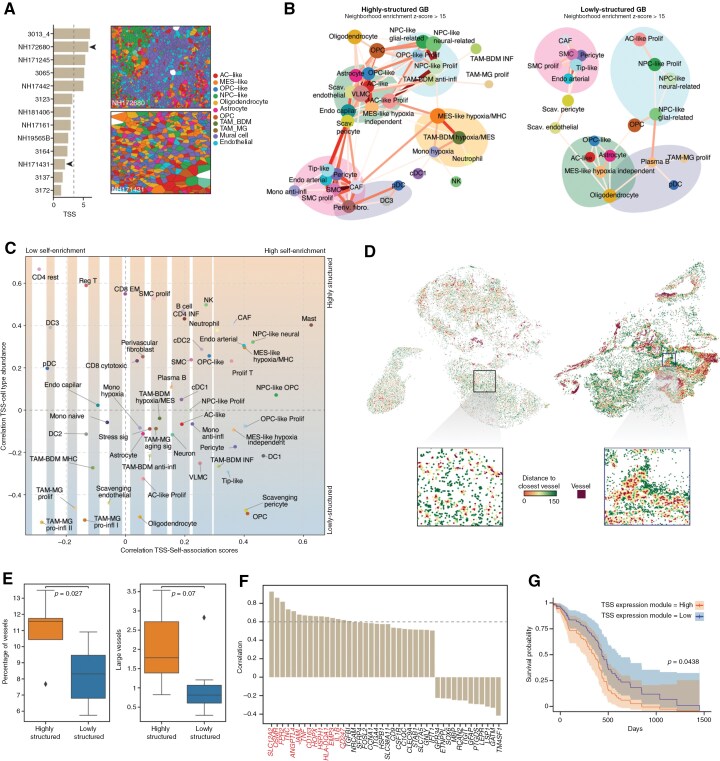
Association of the tumor vasculature in GB organization and structural signature as prognostic. (A) Tumor structure score (TSS; Methods) computed for each GB section (left). Voronoi plots highlight the cell types identified in situ in 2 samples, with cells colored according to the level 3 annotation, described in Figure 2E. (B) Graph representing the neighborhood enrichment between different cell subtypes in space in highly (left) and lowly structured (right) samples independently. Graphs are illustrated in (Figure 5D). (C) The scatter plot displays for each cell type the correlation between its cell type abundance and TSS (y-axis) and the correlation between its self-association score and TSS (x-axis). (D) Spatial visualization of cells associated with vessels across individual samples, with a highly vascularized sample on the right (NH171245), and a lowly vascularized sample on the left (NH181406). Each cell is colored based on its rounded distance to the closest vessel, with a maximum distance threshold of 150 µm. The distance gradient highlights spatial relationships between non-vessel-associated cells and nearby vascular structures. (E) The box plot displays the difference in the percentage of blood vessels and the number of large blood vessels between highly and lowly structured GB. Bars show the standard deviation. The *P*-value was determined using a *t*-test. (F) The bar plot displays the correlation (>0.5 and <−0.4) between the expression of the genes in the RNA-ISS panel and the TSS across samples. Genes with correlation > 0.6 were used to derive a structural GB signature. (G) Kaplan–Meier survival curves comparing overall survival in TCGA-GBMwt patients with low and high module expression levels. The survival analysis adjusts for patient age, sex, and MGMT promoter methylation status. Shaded regions represent 95% confidence intervals for survival probabilities. The *P*-value for the log-rank test is displayed, indicating the statistical significance of differences between the survival curves.

To supplement these findings, we further examined the cellular structure variation between patients and the variations within niche types. While examining the cell maps, we observed that some samples appeared to have more defined structures than others. To quantify this observation, we implemented a metric denominated TSS. The TSS is computed as the mean of the absolute Z-scores derived from the neighborhood enrichment analysis, allowing the classification of samples based on their degree of organization (Figure 6A**and****Figure S8**, Methods). By sorting them based on their degree of organization, we divided tumors into “highly structured” or ‘lowly structured’. Highly structured tumors were organized more clearly and consistently, with most cell types restricted to unique tissue microenvironments. In contrast, lowly structured tumors comprised a patchwork of cells with less observable structure (Figure 6A).

To assess the differences in niche distribution between high- and low-structured samples, we recomputed neighborhood enrichment scores (Figure 6B) by dividing them into separate cohorts based on their TSS status. We observed that: (1) the hypoxic niche was largely absent in the lowly structured tumors, and various hypoxia-related cell types were also absent (MES-like hypoxia/MHC, TAM-BDM hypoxia/MES, Mono hypoxia), (2) CAFs were absent from the vascular niche in lowly structured tumors as opposed to highly structured, and (3) the components of the perivascular compartment differed depending on whether it was highly or lowly structured (**Figure 6B**). For further validation, we assessed the correlation between TSS and cell-type abundance. While low-structured tumors had a greater abundance of scavenging endothelial, oligodendrocyte, and TAM-MG pro-inflammatory cells (Figure 6C), high-structured tumors had a greater abundance of various types of T cells as well as OPC-like and NPC-like neural malignant cells (Figure 6C). Interestingly, MES-like hypoxia independence was close to zero, indicating no correlation between the abundance of TSS. In contrast, MES-like hypoxia/MHC abundance was moderately correlated with higher TSS. To examine this further, we assessed the correlation between TSS and the self-enrichment score of each cell type (Figure 6C**).** This score measures the propensity of a cell from a certain cell type to be surrounded by a cell of its same type compared to random, serving as a quantification of the spatial organization of each cell type. With this, we identified a high correlation between the level of organization of MES-like hypoxia/MHC and MES-like hypoxia independent on the one hand and TSS of each sample on the other, emphasizing the role that MES-like signatures could play in the organization of the tumors.

In addition, we further noted that the abundance of various types of vascular cells was associated with the TSS. To explore this, we first identified vessels within our samples, defined as endothelial and mural cells tightly connected in space. By implementing and applying an algorithm that considers these conditions, we defined the number, size, and composition of vessels present in the tumor (Figure 6D). Examining the distribution of vasculature in the highly and lowly structured tumors, we observed that highly structured tumors tend to have both larger vessels (*P* = .07) and a higher percentage of vessels (*P* = .027) than lowly structured tumors (Figure 6E). With these observations in mind, we set out to investigate the links between TSS and patient outcomes. To start, we computed Pearson’s correlation between the TSS score and the mean gene expression detected on a sample in situ, identifying genes that are highly expressed in structured samples. Next, we established structure-associated expression signatures with the genes presenting a Pearson correlation coefficient above 0.6. Next, we developed a signature indicative of tissue structure by leveraging genes whose expressions correlated with the TSS across samples (Figure 6F). To validate the TSS structure score, we stratified the GB patients in the TGCA cohort^42^ based on the expression of structure-associated expression signature into high and low expression. Then, we computed the Kaplan–Meier estimate for the 2 cohorts to identify the survival differences between both groups. Patients in the TSS high-scoring group saw decreased survival as opposed to the low-scoring group (*P* = .0438; Figure 6G).

In summary, we provide detailed single-cell resolution maps of GB and offer a first glance at not only the relationship between niche composition, TME architecture, and level of organization but also the key components that could impact prognosis.

## Discussion

Our study aimed to comprehensively capture the diverse spectrum of cellular states in GB by gathering data from numerous published cohorts. We acknowledge the difficulty of integrating such datasets with diverse experimental settings such as tissue handling, library preparation method, lab identity, timing, and platform, among others. Following benchmarking of several integration tools, we selected scANVI,^19^ which showed the best balance in removing batch effects while conserving biological variation. This does not exclude that residual batch effects could remain after integration. We tried to mitigate potential confounders in downstream analyses. To generate the final cell label consensus, we did not rely solely on differentially expressed genes to assign cell identity. We complemented the analysis by using Hotspot to reduce the impact of technical variation across batches and identify biologically relevant programs. Beyond well-recognized and recently discovered cell states, we could pinpoint scarce cell populations, such as normal neurons (0,000065%), and novel phenotypes, such as *PLVAP*^hi^ arterial-like ECs and immune-associated scavenging pericytes. Building upon previous studies and the outcome of our analysis, we propose a harmonized annotation in a structured and comprehensive manner (**Figure 2C and****Table S4**).

While the GBmap has been carefully constructed to reduce batch effects and maintain biological relevance, it remains subject to technical and conceptual biases that should be considered when interpreting results. One source of bias arises from integrating datasets with variable experimental procedures—ranging from sample preparation, cell/nuclei isolation methods, and sequencing platforms (10× Genomics, Smart-seq2, etc.) to differences in data preprocessing and normalization. Although our internal benchmarking and tests showed consistent performance, we cannot exclude that lower-quality data or alternative platforms might affect the predictions of certain cell populations or transcriptional programs. Study designs that rely on immune-cell enrichment, or selective sampling could skew cell-type distributions, while residual batch effects across diverse datasets might blur true biological signals. Although nuclei-based profiling typically preserves cell identities, its capture efficiencies and expression biases could still impact prediction accuracy. Using GBmap as a reference, findings should be approached carefully, ensuring validation and further exploration.

By recognizing these potential limitations, one can more accurately leverage the GBmap as a reference resource for cell identity annotation and hypothesis generation, ensuring that subsequent downstream analyses account for these technical and design-driven confounders. We anticipate that the GBmap will “grow” over time as new studies will capture cell (sub)types not yet represented in the core GBmap reference. Ultimately, the GBmap’s comprehensive coverage provides a useful starting point for exploring the cellular complexity of GB, enabling robust cross-dataset comparisons and highlighting the value of careful quality assessment and study design when utilizing large-scale integrated atlases.

To investigate co-localization patterns and the tumor’s architectural features, we used a highly sensitive single-cell ST platform. The limited resolution of previous untargeted spatial transcriptomic studies, either in terms of inability to resolve single cells^16,43^ or in terms of sensitivity,^18^ prompted efforts to address the inability to resolve single cells computationally, either via deconvolution^44^ or via metaprogram analysis.^17^ To address these challenges, we developed consensus transcriptomic signatures from GBmap, enabling our targeted spatial approach to resolve single cells in situ. This validation confirmed the cell state defined in the GBmap and demonstrated its utility for defining common transcriptomic signatures across a highly heterogeneous cohort.

Our analysis revealed 3 modes of spatial organization. Firstly, cell states are self-associated in forming enriched local environments, while others display diffuse distributions, both participating in distinct spatial niches. This highlights how both spatial location and cell distribution regulate cell states. Secondly, specific cell states and functions were confined to particular niches. For example, OPC-like proliferative cells localized to one niche, while non-proliferative OPC-like cells occupied another. Similarly, MES-like hypoxia/MHC cells and MES-like hypoxia-independent cells were found in distinct niches. Thirdly, tumor structure influenced cellular architecture. Consistent with previous studies,^17^ we stratified patient samples into highly and lowly structured tumors. Highly structured tumors featured well-organized vasculature, while lowly structured tumors exhibited less vasculature and lacked hypoxic niches. Interestingly, even nonmalignant cells showed architectural differences between highly and lowly structured tumors, underscoring the interplay between structure and microenvironment.

Finally, we found that tumor structure correlates with patient prognosis. Highly structured tumors, which displayed a well-organized vasculature and pronounced hypoxic niches, were associated with signaling pathways linked to tumor progression, therapy resistance, and sustained angiogenesis, resulting in poorer outcomes in the TCGA cohort (Figure 6G). Notably, genes such as *TNC, ANGPTL4,* and *OSM*, which bulk transcriptomic analyses (as defined by Verhaak et al.^45^) traditionally placed under the mesenchymal subtype, were strongly expressed within these organized, hypoxia-enriched regions. However, our single-cell and spatial data indicate that the mere presence of these MES-like genes does not fully account for patient survival differences. Instead, it is the spatial context, whether or not these genes coincide with distinct hypoxia-dependent niches, that more accurately associates with unfavorable prognosis. Conversely, tumors with low TSS exhibited fewer hypoxic niches yet retained MES-like phenotypes, correlating with better survival than their more structurally organized counterparts. These observations underscore the importance of tumor architecture in shaping GB progression and highlight that the link between a “mesenchymal” signature and clinical outcome is far more nuanced when spatial context is considered.

Our findings refine existing knowledge, emphasizing that GB’s malignant cell states are context-dependent and influenced by the microenvironment. The hypoxic niche appears to be an important factor in glioblastoma progression, warranting further investigation for its clinical relevance. GBmap represents a dynamic framework that supports data mapping, model updates, and exploring novel concepts. Its application illustrates the utility of high-resolution atlases in advancing our understanding of GB biology.

## Supplementary Material

noaf113_suppl_Supplementary_Figures_S1-S8

noaf113_suppl_Supplementary_Materials

noaf113_suppl_Supplementary_Tables_S1-S5

## Data Availability

The newly generated snRNA-seq data (processed and filtered) have been deposited in GEO under accession number GSE211376. The core and extended GBmap (raw and normalized counts, integrated embedding, cell-type annotations, technical and clinical metadata) is publicly available and can be downloaded via cellxgene at https://cellxgene.cziscience.com/collections/999f2a15-3d7e-440b-96ae-2c806799c08c. Files for reference mapping (Azimuth), and transfer learning (core reference model - scArches) were made available at https://doi.org/10.5281/zenodo.6962901. The published datasets that were included in the core GBmap can be accessed under GEO/EGA/SRA accession numbers: GSE103224, GSE131928, GSE138794 (EGAS00001002185, EGAS00001001900, and EGAS00001003845), GSE148842, GSE139448, EGAS00001004422, PRJNA579593, GSE117891, GSE157424, EGAS00001004656, EGAS00001005300, GSE84465, PRJNA588461, GSE135437, GSE163108, and GSE163120. Datasets included in the extended GBmap can be accessed under GEO/links: GSE141946, GSE166418, GSE162631, GSE154795, GSE141383, GSE182109, GSE173278, https://portal.gdc.cancer.gov/projects/CPTAC-3, and https://doi.org/10.17605/OSF.IO/4Q32E. The expression maps and identity of each cell generated in this study using RNA-ISS can be found at https://doi.org/10.5281/zenodo.6954130, and can be interactively explored at an online TissUUmaps viewer https://gbmap-iss.serve.scilifelab.se/. Details of the code and parameters used to create the core and extended GBmap, including downstream analyses, will be available at https://github.com/ccruizm/GBmap. Code used to analyze the spatial transcriptomic datasets is available at https://github.com/Moldia/GBmap_ISS.
