# Supplementary material for: Charting the single-cell and spatial landscape of IDH-wild-type glioblastoma with GBmap
Source: Neuro Oncol. 2025 May 1;27(9):2281–95. doi: 10.1093/neuonc/noaf113 (PMC12526130; doi:10.1093/neuonc/noaf113)
Supplement: noaf113_suppl_Supplementary_Figures_S1-S8 [file noaf113_suppl_supplementary_figures_s1-s8.pdf]

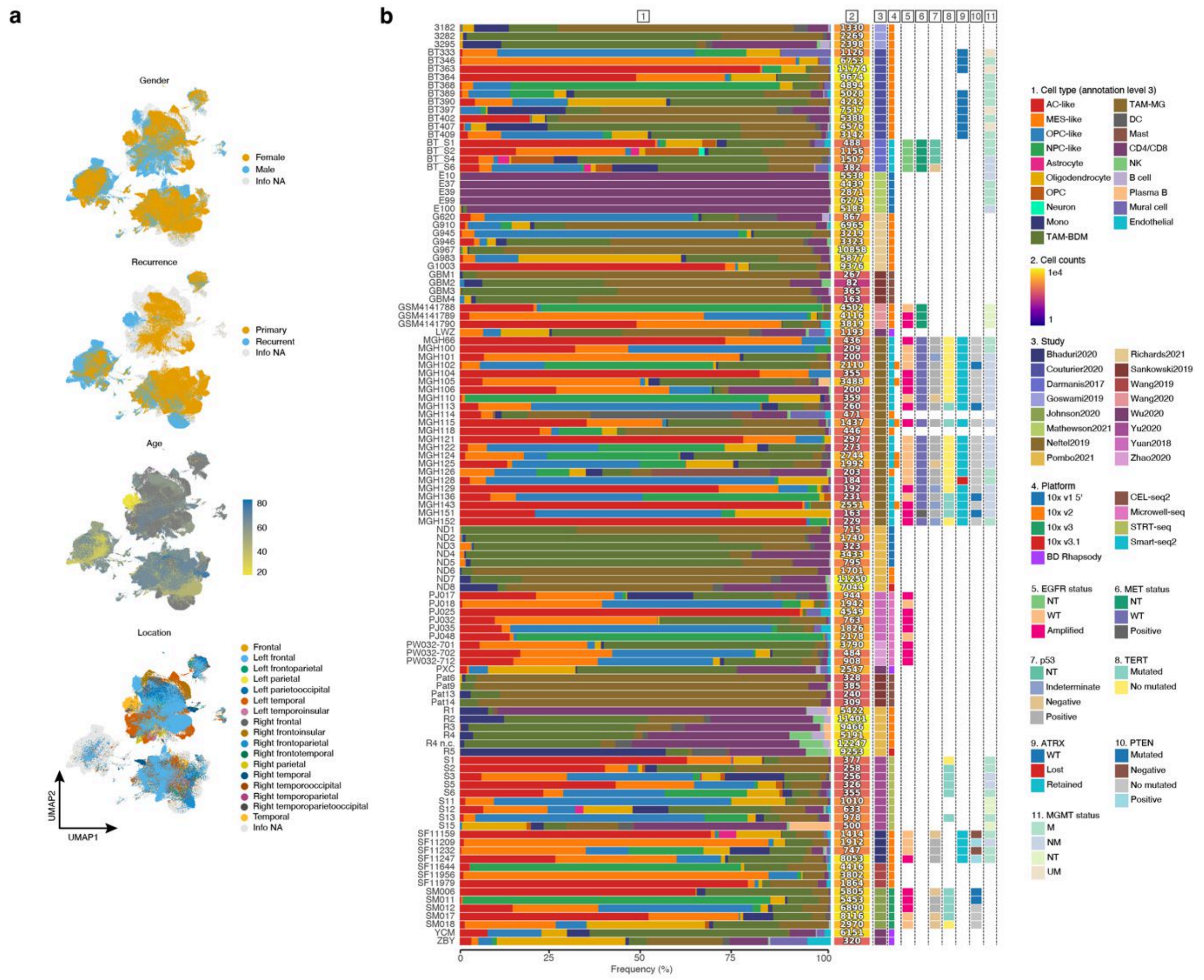

Extended data fig. 1 | GB map features. a, UMAP of the GB atlas colored by different clinical features (gender, recurrence, age, location). b, Bar plot of cell distribution, cell count, study, platform, and genomic features (EGFR, MET, p53, TERT, ATRX, PTEN, MGMT) reported per patient from the subjects included in the core GBmap reference.

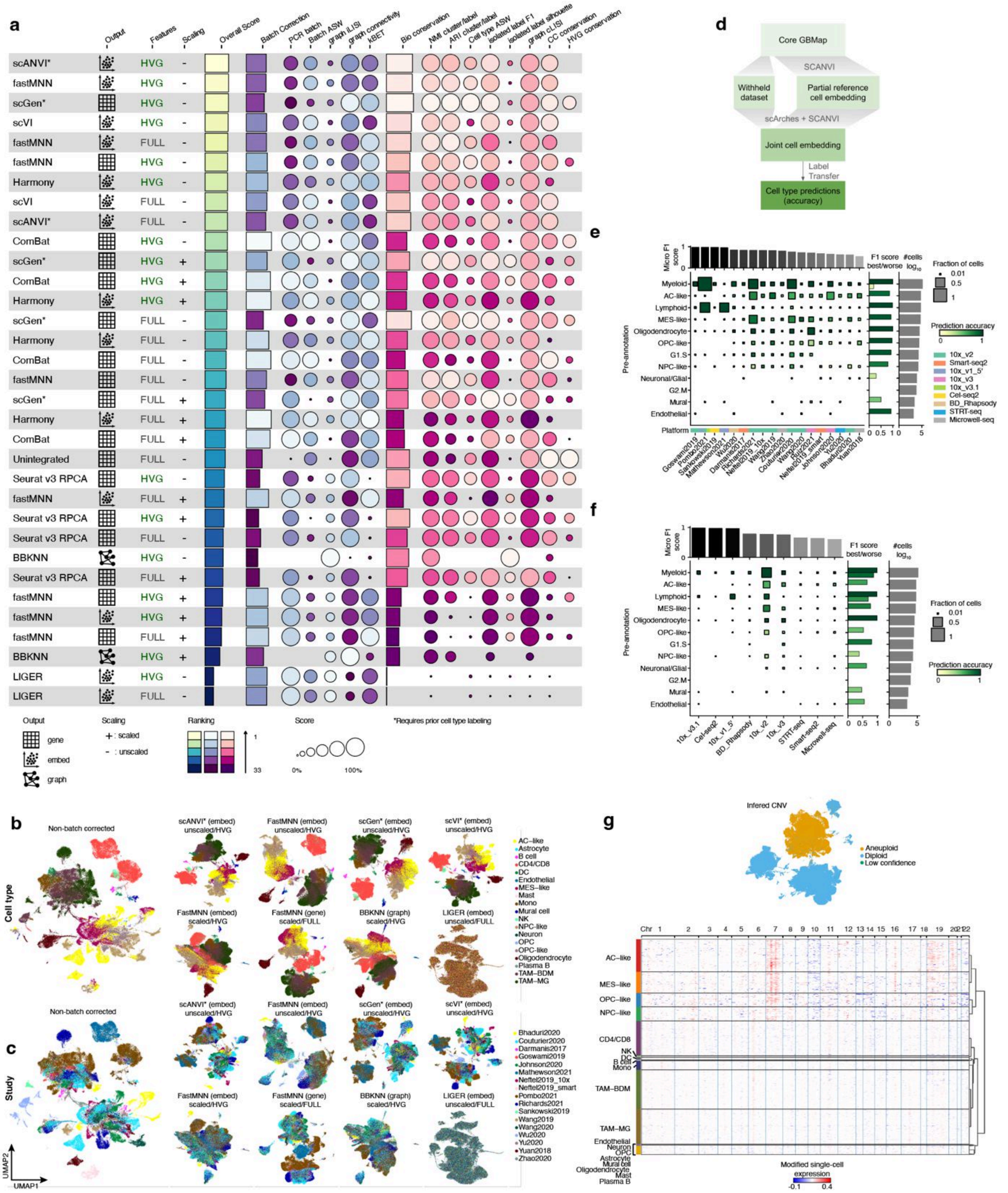
a
Output
Features
Scaling
Overall Score
Batch Correction
PCR batch
Batch ASW
graph iLISI
graph connectivity
kBET
Bio conservation
NMI cluster/label
ARI cluster/label
Cell type ASW
isolated label F1
isolated label silhouette
graph cLISI
CC conservation
HVG conservation
scANVI* HVG -
fastMNN HVG -
scGen* HVG -
scVI HVG -
fastMNN FULL -
fastMNN HVG -
Harmony HVG -
scVI FULL -
scANVI* FULL -
ComBat HVG -
scGen* HVG +
ComBat HVG +
Harmony HVG +
scGen* FULL -
Harmony FULL -
ComBat FULL -
fastMNN FULL -
scGen* FULL +
Harmony FULL +
ComBat FULL +
Unintegrated FULL -
Seurat v3 RPCA HVG -
fastMNN FULL +
Seurat v3 RPCA HVG +
Seurat v3 RPCA FULL -
BBKNN HVG -
Seurat v3 RPCA FULL +
fastMNN HVG +
fastMNN HVG +
fastMNN FULL +
BBKNN HVG +
LIGER HVG -
LIGER FULL -
Output
gene
embed
graph
Scaling
+ : scaled
- : unscaled
Ranking
1
33
Score
0%
100%
*Requires prior cell type labeling
d
Core GBMap
SCANVI
Withheld dataset
Partial reference cell embedding
scArches + SCANVI
Joint cell embedding
Label Transfer
Cell type predictions (accuracy)
e
Micro F1 score
F1 score best/worse
#cells log10
Fraction of cells
0.01
0.5
1
Prediction accuracy
0
1
10x_v2
Smart-seq2
10x_v1_5'
10x_v3
10x_v3.1
Cel-seq2
BD_Rhapsody
STRT-seq
Microwell-seq
Pre-annotation
Myeloid
AC-like
Lymphoid
MES-like
Oligodendrocyte
OPC-like
G1.S
NPC-like
Neuronal/Glial
G2.M
Mural
Endothelial
Platform
f
Micro F1 score
F1 score best/worse
#cells log10
Fraction of cells
Prediction accuracy
10x_v3.1
Cel-seq2
10x_v1_5'
BD_Rhapsody
10x_v2
10x_v3
STRT-seq
Smart-seq2
Microwell-seq
b
Non-batch corrected
scANVI* (embed) unscaled/HVG
FastMNN (embed) unscaled/HVG
scGen* (embed) unscaled/HVG
scVI* (embed) unscaled/HVG
FastMNN (embed) scaled/HVG
FastMNN (gene) scaled/FULL
BBKNN (graph) scaled/HVG
LIGER (embed) unscaled/FULL
Cell type
AC–like
Astrocyte
B cell
CD4/CD8
DC
Endothelial
MES–like
Mast
Mono
Mural cell
NK
NPC–like
Neuron
OPC
OPC–like
Oligodendrocyte
Plasma B
TAM–BDM
TAM–MG
c
Study
UMAP2
UMAP1
Bhaduri2020
Couturier2020
Darmanis2017
Goswami2019
Johnson2020
Mathewson2021
Neftel2019_10x
Neftel2019_smart
Pombo2021
Richards2021
Sankowski2019
Wang2019
Wang2020
Wu2020
Yu2020
Yuan2018
Zhao2020
g
Inferred CNV
Aneuploid
Diploid
Low confidence
Chr
AC–like
MES–like
OPC–like
NPC–like
CD4/CD8
NK
DC
B cell
Mono
TAM–BDM
TAM–MG
Endothelial
Neuron
OPC
Astrocyte
Mural cell
Oligodendrocyte
Mast
Plasma B
Modified single-cell expression
-0.1
0.4

Extended data fig. 2 | Results of dataset integration benchmarking. a, We used a randomly downsampled version of our core GBmap (50% of the cells ~160K) to evaluate the integration methods. Each row corresponds to a tested method, utilizing a specific preprocessing approach. Preprocessing is characterized by "Features," encompassing either "HVG" (Highly Variable Genes) or "FULL" (comprising the selection of 2000 or 5000 most highly variable genes), and "Scaling" (indicating whether gene values were standardized to have a mean of 0 and a standard deviation of 1 across cells). Methods are arranged based on an overall score, which is a weighted average of the batch correction score and the bio-conservation score. The "Metrics" columns have been described in the scIB pipeline. The "Output" column specifies the type of output provided by each method, whether it entails corrected gene counts, an integrated embedding, or an integrated graph. It is worth mentioning that scANVI and scGen received coarse cell type labels as input (semi-supervised methods), as indicated by the asterisk (*). b and c, UMAP plots for the down-sampled version of our core GBmap (50% of the cells ~160K): original unintegrated data (leftmost), top four performing methods (upper rows), and bottom four performing methods (lower rows). The coloration of plots is based on (b) cell identity annotations and (c) batch labels. d, Within GBMap query-to-reference benchmarking. A leave-one-out reference model is generated for each dataset/platform within GBMap. Then, these models are used to predict and assess label-prediction consistency versus out-of-model datasets. e, Square plot highlights an accuracy value retrieved from a query-to-reference mapping task of labeled datasets (x-axis) when queried onto a reference built without them. The y-axis indicates the cell-type classification performance within datasets (rows normalized). The top barplot indicates the overall classification performance of the column dataset, adjusted by cell type numbers. The right bar plots indicate the best and worst accuracies per cell type (greens) and the total number of cells in the full core dataset. Empty squares indicate cases where cell types on a queried dataset are absent in the predictions. f, Same as e, but stratified x-axis by technology instead of datasets.

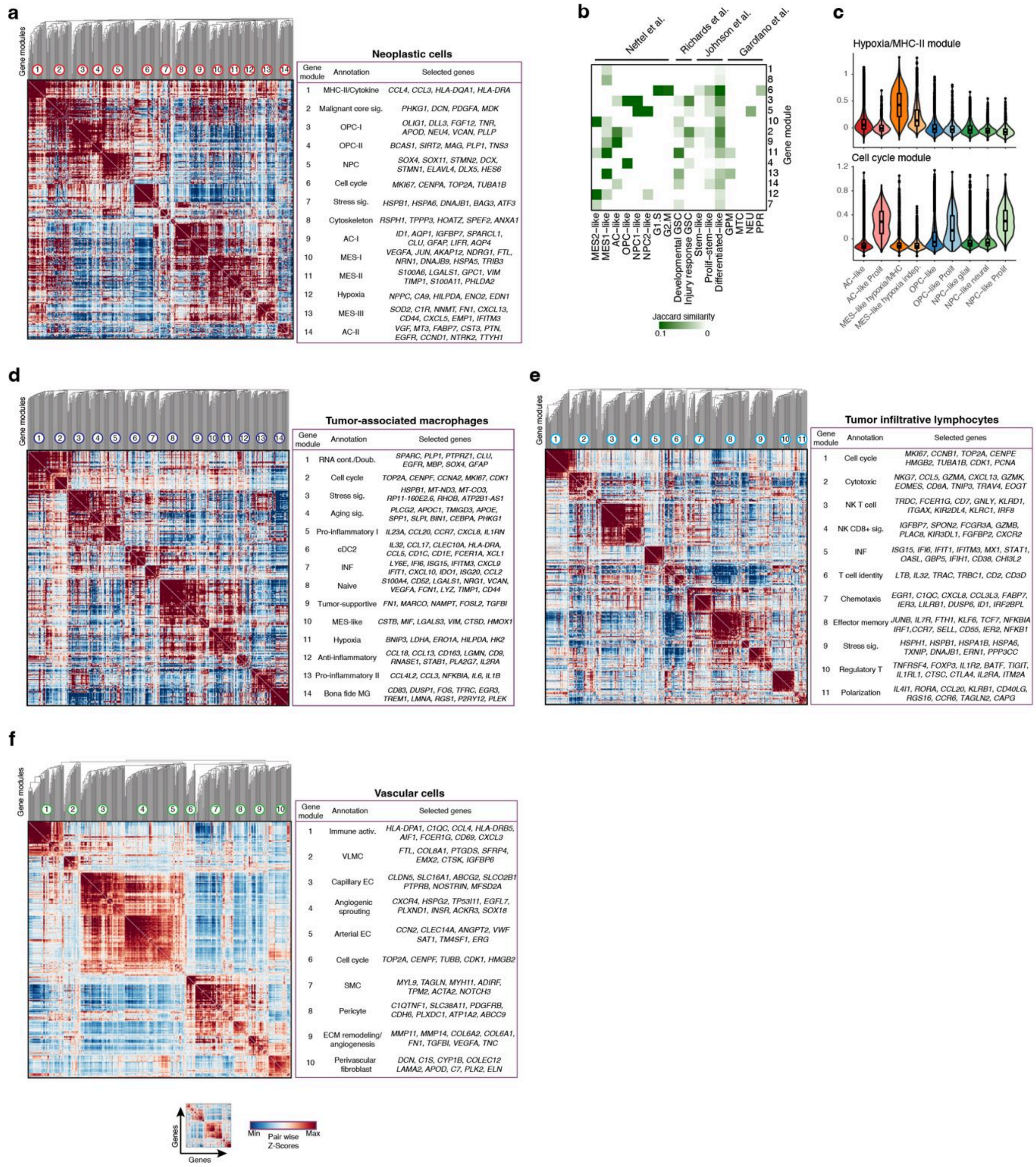

Extended data fig. 3 | Definition of cell states in the GB TME. a, d, e, and f, Heatmap of gene pairwise local correlation (FDR < 0.01) of the top 500 genes detected in neoplastic cells, TAMs, TILs, and vascular cells, grouped into gene modules using Hotspot. On the right, it is the functional annotation and selected genes for each module that helped guide the final cell annotation at the finest level. b, Jaccard similarity comparing published GB signature with our defined malignant Hotspot signatures. c, Module scores of the hypoxia/MHC and cell cycle programs on the cancer cells.

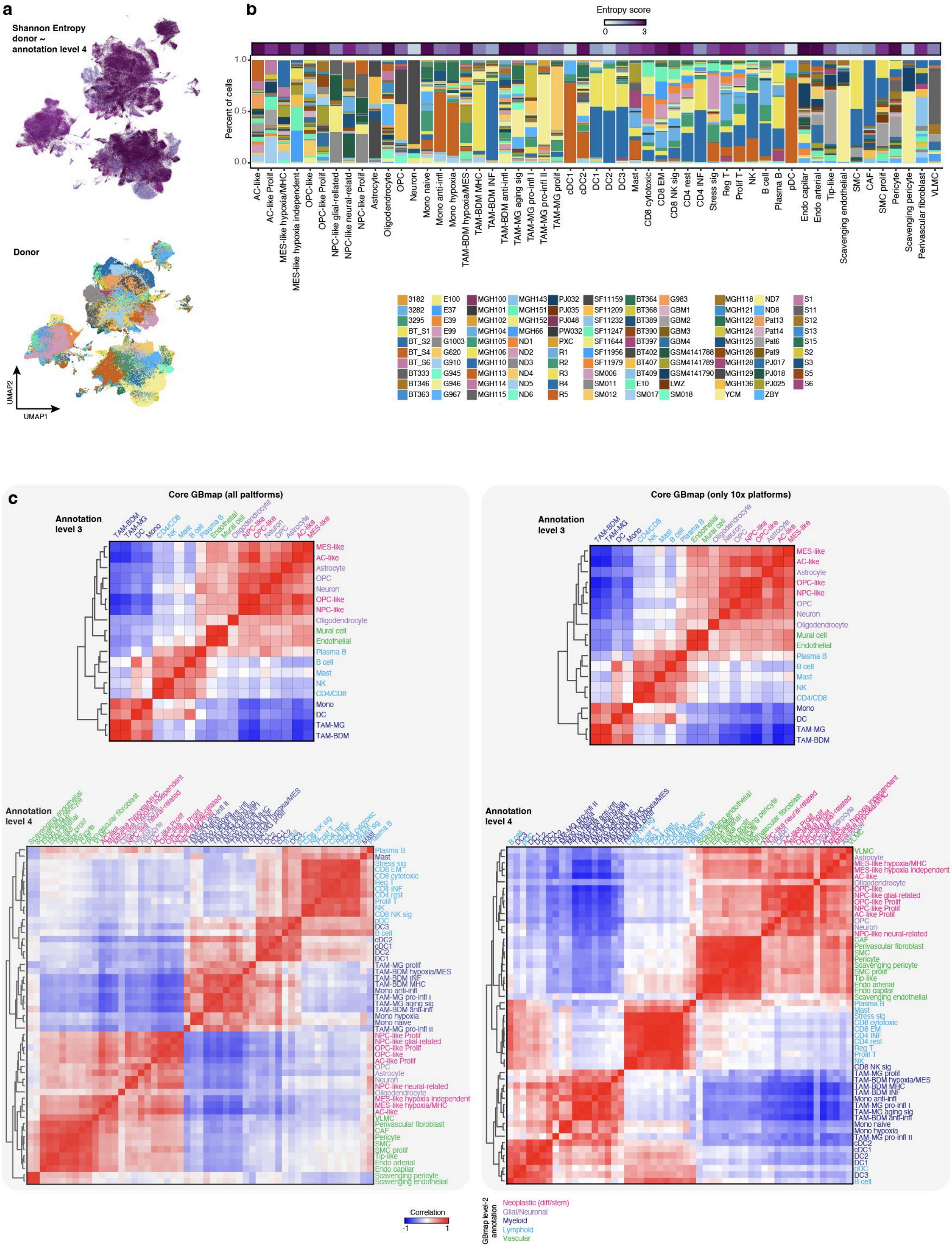

Extended data fig. 4 | Subject diversity in the core GBmap at the finest level of annotation. a, Cells from every cluster (annotation level 4) are colored by the Shannon entropy. High entropy indicates that the cluster contains cells from many different subjects. b, Bar plot shows the subjects' contribution to each cell subtype (annotation level 4). On top, the entropy scores shown in (a). c, Heatmaps illustrating the Pearson correlation coefficients among various cell (sub)types (level 3 and 4)

found within the core GBmap and the version comprising solely 10X Genomics datasets. The heatmap's color scale represents the strength of Pearson correlation, ranging from no correlation (depicted in dark blue) to highly correlated (depicted in dark red). Cell (sub)types are color-coded according to a broader level of annotation (GBmap annotation level 2).

type (annotation level 4). It shows an overall agreement between the malignant GB signatures defined by Neftel et al. and the GBmap labels.

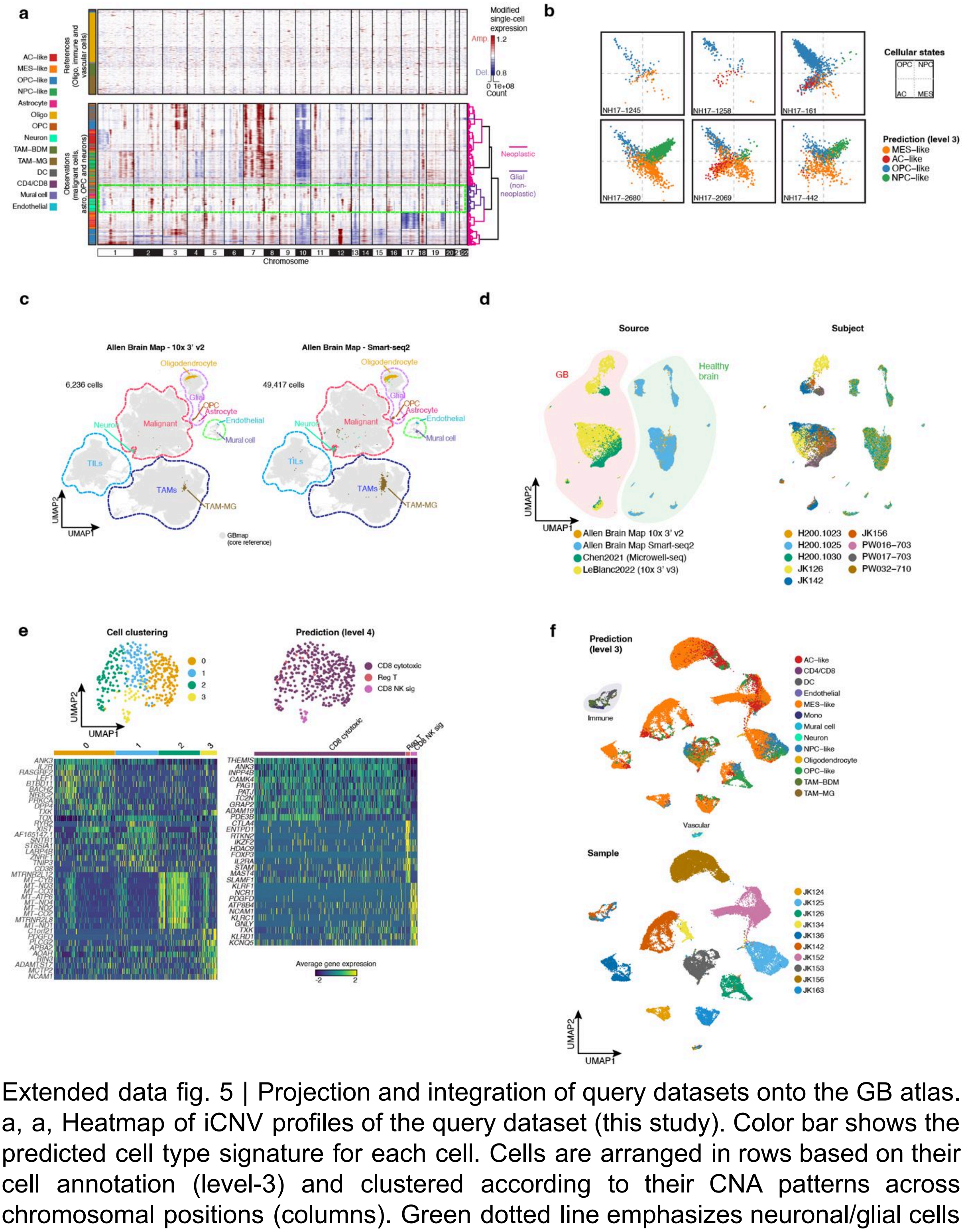

Extended data fig. 5 | Projection and integration of query datasets onto the GB atlas. a, a, Heatmap of iCNV profiles of the query dataset (this study). Color bar shows the predicted cell type signature for each cell. Cells are arranged in rows based on their cell annotation (level-3) and clustered according to their CNA patterns across chromosomal positions (columns). Green dotted line emphasizes neuronal/glial cells lacking iCNV. b, Two-dimensional representation of cellular states (cancer cells) from selected GB samples from Ruiz2021, colored by GBmap predicted cell type (annotation level 3). c, Projection of human cortex brain cells from the Allen Brain Map initiative (https://portal.brain-map.org) onto the core GBmap UMAP. d, UMAP of the in silico mix of human cortex brain cells from the Allen Brain Map initiative and

six GB samples from published studies (three samples from Chen et al. 2021. and three from LeBlanc et al. 2022.), colored by dataset and subject. e, UMAP of reclustered TILs from de novo GB dataset (11 samples processed in this study) and scaled gene expression for each subcluster. The left side shows the unsupervised annotation, and on the right, the predicted cell states are based on the finest annotation of the GBmap (level-4). f, UMAP clustering of the LeBlanc et al. 2022 dataset includes only data derived from patient-derived explants (PDEs). On the bottom, UMAP is colored by predicted cell type (annotation level 3).

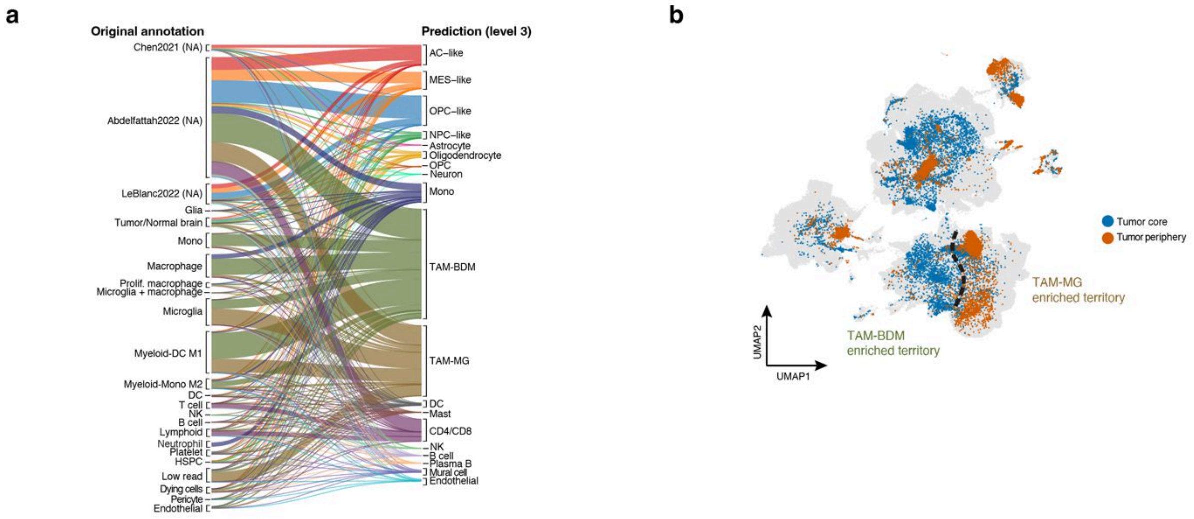

Extended data fig. 6 | Projection and integration of query datasets onto the GB atlas. a, Comparison of original annotation with predicted cell types (level-3 annotation) after label transfer from the GBmap core. b, UMAP displays the distinction between core and periphery regions of multisector biopsy studies included in the GBmap. The dashed line indicates the estimated boundary between the TAM-BDM and TAM-MG territories.

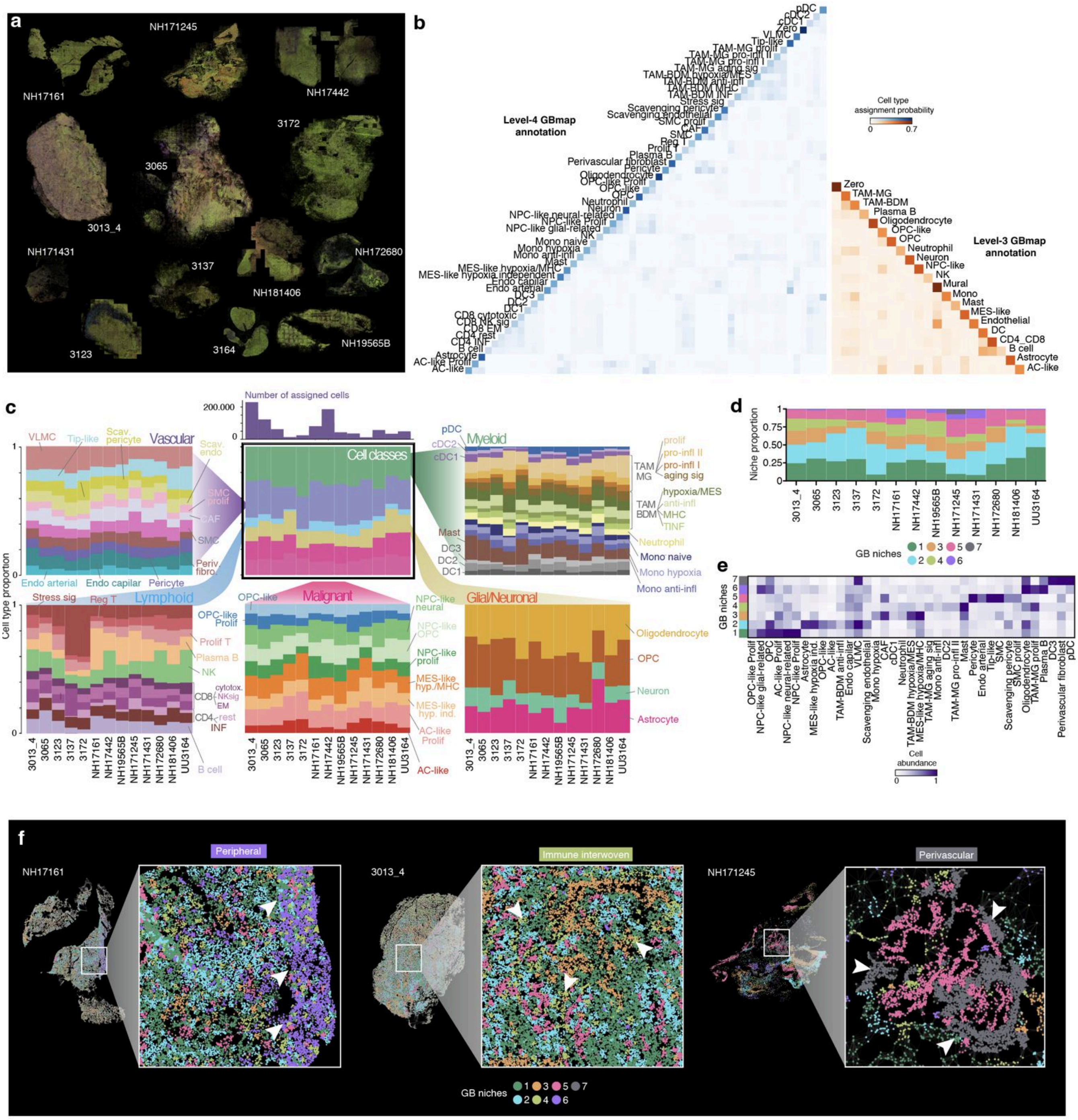

Extended data fig. 7 | Single-cell spatial mapping of GB using RNA-ISS. a, Molecular maps of the 13 GB sections profiled by RNA-ISS. Colors of individual spots represent the identity of individual reads detected. b, Heat map illustrating the mean probability of assignment of each cell to a specific (sub)type using pciSeq for level-4 (left) and level-3 (right) GBmap annotations. c, Stacked bar plot representing the relative abundance of the cell classes identified on each samples by pciSeq (center) and the relative frequency of cell types included on each class. A small bar plot illustrating the amount of profiled cells on each sample is included on top of the central stacked bar plot. d, Relative niche abundance of each sample, represented as a stacked bar plot. e, Heatmap representing the relative abundances of cell subtypes by niche. Only cell types with a maximum relative abundance above 0.3

are represented. f, Representative GB sections showing the distribution (arrows) of the peripheral (left), immune interwoven (middle), and perivascular (right) niches. The niches identified are represented by coloring individual cells (nodes) by the niche they have been assigned to. Edges connect each cell with their closest 15 cells, used to define the composition of their niche.

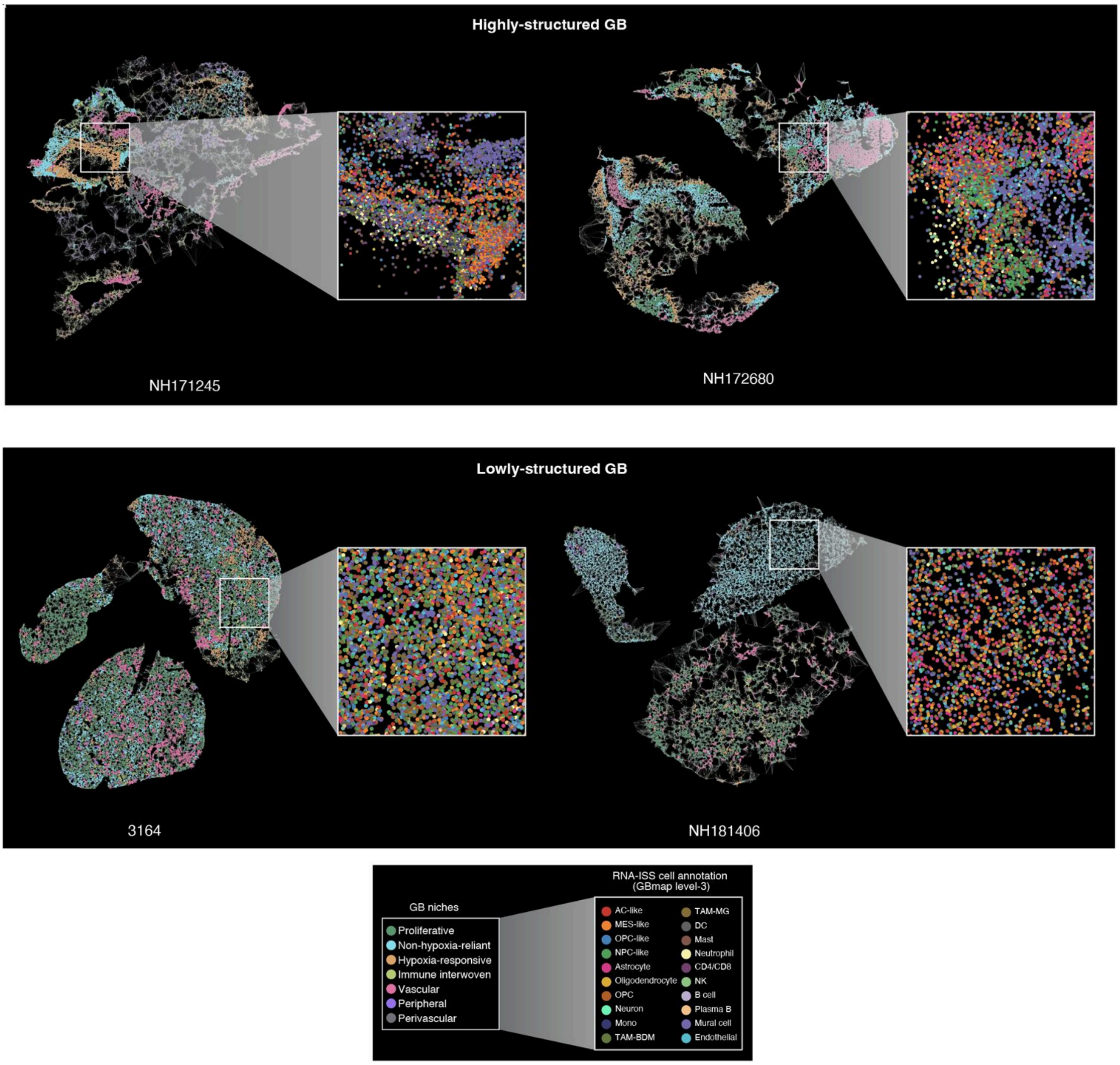

Extended data fig. 8 | Highly- and lowly-structured GB. Niches identified in the GB sections profiled with RNA-ISS. Niches identified in four sections are represented by coloring individual cells by the niche assigned to them on highly- (top) and lowly-structured samples (bottom), as defined based on their TSS score. Edges connect each cell with its closest 15 cells, used to define the composition of its niche. Zoom-in displays the cell identities defined by pciSeq based on the harmonized annotation in the GBmap (level-3).
