# Supplementary material for: Charting the single-cell and spatial landscape of IDH-wild-type glioblastoma with GBmap
Source: Neuro Oncol. 2025 May 1;27(9):2281–95. doi: 10.1093/neuonc/noaf113 (PMC12526130; doi:10.1093/neuonc/noaf113)
Supplement: noaf113_suppl_Supplementary_Materials [file noaf113_suppl_supplementary_materials.docx]

**Methods**

**Tissue Collection and Preparation**

GB tissue sections from primary resections of adult patient tumors were obtained via Dr. Sebastian Brandner as part of the UK Brain Archive Information Network (BRAIN UK), funded by the Medical Research Council. Relevant ethical consent was provided by BRAIN UK (Ref:19/005). Fresh tumor tissue was collected and frozen immediately. After embedding in OCT, 20 µm sections were cut from the OCT blocks and mounted on positively charged slides (VWR superfrost Plus slides). The slides were then stored at -80° C until use. Additionally, GB sections from the Uppsala-Umeå Comprehensive Cancer Consortium (U-CAN) biobank were incorporated. These sections, sourced with patient consent, underwent histopathological selection (>60% tumor cells). For spatial transcriptomics analysis, 10 µm sections of fresh frozen tumor tissue within OCT blocks were used. Slides were prepared as mentioned earlier and stored at -80°C. Ethical approval was obtained from the Ethical Review Board of Uppsala, Sweden (Dnr 2007/353 with addenda) (Uppsala Biobank no: 827-2014-087, U-CAN: 2014-004), aligning with the Declaration of Helsinki.

**Single-nuclei RNA sequencing**

Nuclei isolation was performed on 10-20µm cryosections for each specimen. The tissue slices were placed on 500µl of Nonidet P40 with salts and Tris (NST) lysis buffer and homogenized on ice using a glass-on-glass Dounce homogenizer with ten strokes using the loose pestle, followed by 15 strokes of the tight pestle and incubated for 5 min on ice. Nuclear homogenates were filtered through a 70µm Flowmi cell strainer (Bel-Art) and centrifuged for 5 min at 500g, at 4 °C. The pellet was resuspended in wash buffer1 and filtered with a 40µm Flowmi cell strainer (Bel-Art). Nuclei were stained with DAPI 1:200 dilution (2µg/ml stock concentration; Sigma-Aldrich) 5min at room temperature (RT) before sorting. Fluorescence-activated nuclei sorting was done on a Sony SH800 cell sorter (Sony Biotechnology) using a 100µm nozzle. Single Cell Gene Expression 3' v3 (10x Genomics) was used for single-cell/nuclei capturing and library construction, as described in the Genomics Single Cell RNA Reagent Kits User Guide. Briefly, 15,000 single sorted nuclei were loaded into a channel of a Chromium Single Cell Gene Expression 3' Chip. Single nuclei were partitioned into droplets with gel beads in the Chromium, followed by barcoded reverse transcription of RNA, cDNA amplification, fragmentation, and sample index ligation. The quality of the libraries was assessed on a 2100 Bioanalyzer (Agilent) and sequenced on a NovaSeq (Illumina).

**Gene Selection for RNA-ISS**

The first step to perform RNA-ISS experiments was to select a limited set of genes that, when profiled, could be used to assign a cell type identity to each profiled cell. By selecting genes representative of the full range of states in the GBmap. For this gene panels were assembled by selecting cell type markers based on the sn/scRNA-seq data included in the extended GBmap using Scanpy^1^ and Spapros^2^ and further curated manually via literature assessment and gene expression constraints. Briefly, we selected the top 500 genes via pairwise local correlation detected in malignant, myeloid, lymphoid and vascular lineages within the Core GBMVerse dataset. We then excluded genes that had low overall expression in the dataset as well as genes that saw high expression across all cells, as being unsuitable for distinguishing the annotated cell states. This left us with 310 genes for 49 annotations identified, which were then further supplemented with genes identified in literature, genes identified via top differential expression (DE) analysis, and via the Spapros pipeline. To ensure minimal confusion of malignant cells with nonmalignant cells, we selected top DE genes via the rank_genes_groups function in Scanpy for each annotation level in the Core GBMap. Each gene was then scored for average expression for each annotation level using the score_genes function in Scanpy, and selected based on specific expression within target annotations. Finally, after selecting genes that high overall scores across annotations, we validated the expression of the same genes in the Extended GBMap. A total of 209 genes composed the final gene panel, encompassing malignant (OPC-like, AC-like, NPC-like, and MES-like) and non-malignant cells (microglia, macrophages, oligodendrocytes, astrocytes, neurons, endothelial cells, T cells, dendritic cells, and mural cells), as well as signaling markers of interest. For these genes, padlock probes were designed.

**Probe Design for RNA-ISS**

To facilitate the streamlined design of reagents for RNA-ISS, we have developed a user-friendly Jupyter notebook available at our GitHub repository (https://github.com/Moldia/PLP_directRNA_design). This notebook offers a systematic guide for executing the following tasks: starting with a list of gene IDs and a reference transcriptome, we extract the mRNAs corresponding to all described isoforms of each gene. These extracted sequences are then aligned using CLUSTALW2^2^, and common regions are segmented into all possible 30-mers (e.g., nt 1-30, 2-31, and so on). These resulting 30-mers undergo successive filtering stages to identify optimal targets for padlock probe binding. Initially, a GC content filter retains 30-mers within a specified range (50-65%). Subsequently, 30-mers containing a C or G at position 16 are retained due to T4RNAl2's favorable efficiency towards 3' terminal G or C. Non-overlapping, suitable 30-mers are subjected to specificity assessment, using Cutadapt software with allowance for up to 6 mismatches against the transcriptome. 30-mers displaying non-specific hits—those not associated with the query gene—are excluded, ensuring the absence of off-target effects. From the pool of 30-mers passing the specificity check, a subset (typically 5) is chosen, and the final step involves generating distinct padlock probe sequences with unique barcodes linked to individual genes. These custom-designed probes are ordered as DNA Ultramers from Integrated DNA Technologies (IDT), featuring a 3' terminal RNA base, synthesized at a 4nmol scale, and resuspended in IDT buffer at a concentration of 200 uM.

**RNA-ISS experimental methodology**

Tissue sections were thawed and allowed to reach room temperature for 5 minutes. The sections were then fixed in 3% formaldehyde for 5 minutes at room temperature. The fixative solution was removed, and the samples were washed three times with PBS (room temperature). We then permeabilized the sections with a 0.1 HCl incubation for 5 minutes, followed by two washes in PBS. The samples were progressively dehydrated with a 70% ethanol bath for 2 minutes, followed by 100% ethanol bath for 2 minutes, then air dried. We attached secure-seal chambers to cover the samples and filled the chamber with PBS-Tween 0.5%, followed by a PBS wash.

A probe solution was prepared using the following recipe: 2x SSC, 10% Formamide, and 10 nm of each padlock probe, and incubated on the samples overnight at 37 C. The next days, we washed the unhybridized excess probes with two washes of 10% formamide in 2x SSC, followed by two washes in 2x SSC. After removing the last SSC wash, a ligation mix was prepared as described in the "Probe ligation" step in the protocol (<https://www.protocols.io/view/home-made-direct-rna-detection-kqdg39w7zg25/v1>) and incubated on the samples for 2 hours at 37 degrees Celsius. After ligation, the samples were washed twice with PBS, and an amplification mix was prepared as in the "Amplification of the padlock probes" step in the linked protocol. The amplification reaction was carried out overnight at 30 C. The next day, the samples were washed three times with PBS, and L-probes (or bridge probes) were incubated for 30 minutes, as described in the step "hybridization of L-probes" in the linked protocol. Excess probes were washed out with two washes in 2x SSC, and detection oligos and DAPI were incubated for 30 minutes as indicated in the protocol. Excess detection oligos were washed out with two washes in 2x SSC. TrueBlack was applied to quench background fluorescence, if necessary, according to the manufacturer's instructions. Samples were mounted in SlowFade gold, and cyclical imaging was performed. After each imaging cycle, L-probes and detection oligos were stripped with two washes of 3 minutes in 100% formamide, followed by five washes in 2x SSC. The hybridization of L-probes and detection oligos for the following detection cycle was performed as above.

**Imaging**

Imaging was performed using a standard epifluorescence microscope (Zeiss Axio Imager.Z2) connected to an external LED source (Lumencor® SPECTRA X light engine). The light engine was set up with filter paddles (395/25, 438/29, 470/24, 555/28, 635/22, 730/40). Images were obtained with an sCMOS camera (2048 × 2048, 16-bit, ORCAFlash4.0 LT Plus, Hamamatsu), automatic multi-slide stage (PILine, M-686K011), and Zeiss Plan-Apochromat objectives 20x (0.8 NA, air, 420650-9901), 40× (1.4 NA, oil, 420762–9900). Filter cubes for wavelength separation included quad-band Chroma 89402 (DAPI, Cy3, Cy5), quad-band Chroma 89403 (Atto425, TexasRed, AlexaFluor750), and single-band Zeiss 38HE (AlexaFluor488). Each field-of-view (FOV) was imaged with 21 z-stack planes with 0.5 μm spacing and 10% overlap between FOVs.

**Processing of published data and construction of the core GBmap reference**

For the construction of the core GBmap, only samples confirmed to be GB, IDH-wildtype (based on the clinical metadata provided in each study), and containing at least 1000 cells were included. Transcriptomic data coming from nuclei were not considered for the training of the reference model. The 16 datasets collected in the core GBmap (**Supplementary Table 1**) were obtained either as raw or TPM normalized (for Smart-seq2 studies) count matrices. In studies where raw count matrices were unavailable (Couturier2020, Bhaduri2020), BAM files were converted to FASTQ files and re-aligned to GRCh38 using CellRanger v3.1/4.0. All gene names were updated to the latest HUGO nomenclature using HGNChelper^3^. All clinical/diagnostic metadata was harmonized and preserved.

Before integrating the datasets, we applied homogeneous filtering parameters to include high-quality cells, excluding cells that expressed fewer than 500 genes, 1000 UMI counts (for datasets where applicable), and more than 30% mitochondrial reads. We estimated and discarded potential doublets for each droplet-based dataset using DoubletFinder^4^. To determine which approach could find the best balance between reducing technical variance caused by sequencing platforms and retaining biological information, we evaluated nine different batch integration tools (BBKNN, scGen, ComBat, fastMNN, Harmony, scANVI, scVI, LIGER, and Seurat v3 RPCA) using a scIB benchmarking pipeline^5^ with default integration parameter settings. To enhance computational efficiency, benchmarking was conducted on a subset of the total atlas (50% of the cells in the core reference). This subset encompassed data from 109 donors and a total of 167,878 cells. All benchmarked methods were executed twice, once using the 2,000 most highly variable genes (HVGs) and again with the 5,000 most HVGs. For methods not requiring raw counts as input, they were executed twice on each gene set: first with gene counts scaled to have a mean of 0 and standard deviation of 1, and second with unscaled gene counts. Two integration methods, scGen and scANVI, permitted the input of cell-type labels to guide the integration process. To harmonize cell type labels from different sources, we annotated each dataset using automated and manual methods^6^. For the automatic cell annotation, we compiled a curated gene marker list from various studies (**Supplementary Table 2**). We provided a signature for the cell types part of the GB TME and used it as input for the Cell-ID algorithm^7^. After, for the manual assignation of cell identity, we considered the results from the automatic cell annotation, the original cell label (when available), and cell-type annotation available on the TISCH website^8^ (when available), and the expression of cell type-specific marker genes identified using the Wilcoxon rank-sum test by comparing all cells within a specific cluster to all cells outside said cluster. Particularly for the accurate identification and annotation of neoplastic cells, for all immune-enriched datasets, CNV inference was carried out using the SCEVAN package^9^, classifying cells that were either diploid or aneuploid. This preliminary coarse cell type labeling was used for the model training and integration, and the 'study’ was designated as the batch parameter. The quality of each integration was assessed using 12 metrics, with four gauging batch correction quality and eight quantifying the preservation of biological signal after integration. Overall scores were computed by taking a weighted mean (0.4:0.6) of batch effect removal and biological variation conservation. Methods were ranked based on their overall score.

Based on the metrics used to evaluate the methods, scANVI^10^ was the top-scoring integration method. We used scANVI under the transfer-learning model implemented in the single-cell architectural surgery algorithm^11^ (scArches). The pipeline was run on the raw counts of the 5000 most highly variable genes (HVGs), using ‘study’ as the batch variable and the recommended parameters of the tool. The pipeline output is the latent representation of the integrated data that serves as input for clustering and dimensional reduction visualization. We used a k-nearest neighbor graph (k-NNG)-based Leiden clustering^12^ to detect the distinct cell populations and Uniform Manifold Approximation and Projection (UMAP)^13^ for data embedding and two-dimensional reduction. UMAP visualization of the core GBmap (**Figure 1b**) was generated using the plot1cell package^14^. Total count normalization was done by initially dividing each count by the total count per cell and multiplying by 10,000, followed by log transformation using a natural *log*(*X* +1).

After co-embedding all cells, we refined the cell annotation by manually assigning a cell identity to each cluster, considering our unified preliminary cell annotation and the expression of specific marker genes that correctly defined each broad cell type/state (level-1, -2, and -3 annotation). To determine the next level of cell identity (level-4 annotation), we sub-selected, re-clustered, and identified the primary axes of transcriptional variation on each cell territory of interest (malignant, lymphoid, myeloid, and vascular) using Hotspot^15^, which allowed the identiﬁcation of genes that vary in a contextualized fashion. The output is the organization of the genes of each territory into co-varying groups (module/program). We ran the tool using the latent space inferred by scVI^16^ to build the k-NNG and used it as input of the source of cell-cell distances to compute the Euclidean distance in the low-scVI-dimensional space. Hotspot employs a negative binomial distribution to compute pair-wise local correlations between the top 500 lineage autocorrelated genes and group them into correlated modules. Each gene module was matched with a thorough literature search of specific phenotypes that delineated a given cell state. These included findings from previous studies in high-grade gliomas or extended to other cancer types if there was no match in other brain-related pathologies. After calculating the enrichment of each cell for every module, the phenotype assignment was performed based on the highest cell score for a given gene program.

To measure the degree of patient contribution to each cell phenotype (diversity of donors in a cluster) at the higher resolution annotation (level-4), we calculated Shannon's Entropy of the subjects per (sub)cell type. To identify clusters with low donor entropy, we calculated the label entropy for a cluster in which 95% of the cells originated from a single donor, while the remaining 5% of cells were distributed across all other donors. Clusters with donor entropy below 0.19 were classified as clusters with low donor entropy.

**Data processing of *de novo* GB samples**

Raw BCL files generated by the sequencer were demultiplexed using Cell Ranger mkfastq (v3.1.0) to generate the FASTQ files. Each sample was mapped to the human reference genome (GRCh38 v3.0.0) provided by 10x Genomics using the Cell Ranger count with default parameters to obtain the gene count matrix. For single nuclei samples, the reference for pre-mRNA was created using the manufacturer's guidelines (<https://support.10xgenomics.com/single-cell-gene-expression/software/pipelines/latest/advanced/references>).

**Query projection onto the GBmap core**

For the projection and annotation of external data sets onto the GBmap, we first created an object that could be manipulated and utilized by the Azimuth algorithm part of the Seurat package^17^. We imported the Anndata object into R and created a Seurat object that contained the raw and normalized count matrix, cell metadata, and UMAP embedding. The Azimuth label-transferring method uses the low-dimensional structure of the reference and finds 'anchor' genes on the query dataset that enables the projection and transfer of the cell annotations between data sets. For the eleven patients profiled by snRNA-seq in our study, we used unsupervised anchoring based on the first 50 principal components and the log-normalized matrices. Each cell in the query dataset gets a prediction probability, and the cell assignment is performed based on the highest score for a given cell type/state. To confirm the correct assignment of neoplastic and non-neoplastic cells, we used the inferCNV package^18^, placing as reference cells that were not expected to carry any CNV, such as immune and vascular cells. Detection of marker genes among predicted cell types and states was done using a Wilcoxon rank-sum test.

**Expansion of the GBmap by transfer learning**

To extend the GBmap, we gathered raw count matrices from newly generated public datasets available after the curation of the core reference atlas (April 2021) (**Supplementary Table 1**). By using the deep learning scArches algorithm^11^, the core GBmap can allow re-annotation of queried cells, update the currently trained model, and capture differences that could represent new cell types or states. In short, scArches trains adaptors that are added to the reference embedding model, which facilitates the creation of a de novo joint embedding between the new datasets and the core GBmap, enabling the generation of a new dimensional reduction and clustering and re-analysis of the extended reference. The new datasets were QC filtered using the same parameters previously established for constructing the core GBmap. We merged the data matrices of the different studies and selected the same 5000 HVGs employed to train the reference model. Raw counts were used as input for scArches. The integration pipeline was run to 're-train' adapter weights, thus enabling the mapping of new query data into the core GBmap dimensional embedding. To find DEGs between cell types, we used a Wilcoxon rank-sum test comparing cells in the core reference with a specific cluster from the updated version of the GBmap.

**Within core GBmap Leave-One-Out Analysis**

In this step, we systematically withhold one dataset at a time from core GBmap. The withheld dataset serves as the query dataset while we generate a partial reference using the remaining data from the GBmap core. This partial reference is built as a scANVI model, with "author" as a batch key and "celltype_assigned" as a covariate key for semi-supervised training. Using this reference, we perform a query-to-reference label transfer from the constructed partial reference to the held-out dataset using scArches model for 500 epochs with no weight decay (max_epochs = 500, plan_kwargs={‘weight_decay’: 0.0}, check_val_every_n_epoch=10). We compare the transferred labels to the pre-annotated cell type labels across two cell type annotation hierarchies and report the accuracy of the model predictions, as well as limitations in GBmap annotation-label generalization. A similar analysis was performed, withholding one technology at a time. Here, “platform” was used as a batch key and "celltype_assigned" as a covariate key for semi-supervised training of scANVI when constructing a partial reference dataset. Other steps of the analysis mirror the procedure described above.

**Mapping Unseen Datasets from the Extended GB Dataset onto the Core GBmap**

In this step, we map two datasets (Ruiz_2021 and Xie_2021 from extended GBmap, with newly independently generated cell type labels, onto the reference core GBmap. Similarly, we use the scANVI model with "author" as the batch key and an "annotation_level_3" key as a covariate for training the models (scANVI and scArches) with the same parameters as for the leave-one-out analysis. As in our current work, we compute per-cell type accuracies of the label transfer to estimate the generalization of GBMap in external cell-type prediction, as well as per-cell type errors.

**Image processing and decoding of RNA-ISS data**

Each FOV image was maximum intensity projected to obtain flattened two-dimensional images. These images were then analyzed with in-house custom software. Each two-dimensional FOV was exported, aligned between cycles, and stitched together using the MIST algorithm. Stitching was followed by retiling to create smaller, non-overlapping 6000x6000 pixel images that were then used for decoding. The decoding pipeline can be found on the Moldia GitHub page (<https://github.com/Moldia/iss_starfish/>). Using Starfish, images were initially filtered using a white top hat filter. The filtered images were subsequently normalized, and spots were detected using the FindSpots module from Starfish and decoded using the MetricDistance module. Finally, the resulting spots were screened based on the distance to the closest expected barcode in the reference codebook.

**Cell typing and spatial statistical analysis of RNA-ISS**

After decoding the identity of individual reads in the profiled samples, cell segmentation was performed. For this, DAPI stainings, imaged together with the ISS cycles, were used to identify and segment individual nuclei using Stardist^29^ as a segmentation algorithm. After segmenting individual nuclei across sections, we aimed to assign reads to individual cells. For this, we employed probabilistic Cell Typing (pciSeq)^19^, which performs a probabilistic and iterative assignment of reads to cells while identifying the cellular identity of cells based on the prior expression signatures of each cell type. For this task, cell type-specific reference expression signatures were defined based on the GBmap annotations (level 4), computing the mean expression signature of all the cells assigned to each cell type. Using this approach, each cell was given a probability of belonging to each pre-defined cell type. This algorithm also includes the possibility of assigning profiled cells to a “Zero” class, which corresponds to cells presenting either a too-low expression or missing the required markers to be assigned to any of the predefined cell types. Cells presenting the highest assignment probability in this category were excluded from further analysis.

After probabilistic cell typing, cells identified presented both an expression signature, a spatial localization, and a probability of belonging to each cell type identified in the GBmap (core). To simplify the downstream analysis, the cell type presenting the highest probability for each cell profile was used as its predicted cell type (level 4 in the GBmap). Furthermore, a second cell type annotation, corresponding to annotation level 3 in the GBmap, was given to each cell based on the correspondence between level 3 and level 4 annotation of the GBmap (i.e. cells considered as “AC-like prolif” in level 4, were considered “AC-like” in level 3). See Figure 2c for reference.

To explore the cellular environment of each cell and define cellular niches, each cell was re-defined based on the local neighborhood of each profiled cell. Cells situated within 40um close to each cell were considered, creating a cell-by-neighboring cell types matrix. Essentially, for every cell, we quantified the number of cells of each cell type present in its defined neighborhood, as done by Kukanja et al 2024^27^. Cell-by-neighborhood matrices were then preprocessed following standard single-cell preprocessing steps, including library size-based normalization and log-transformation of the counts. Graph-based clustering was performed using Leiden clustering to define cellular niches. Niches were further represented via UMAP low dimensional representation. Clusters resulting from this process represented tissue niches, defined as groups of cells that present the same local microenvironment. Since many cellular niches were sample, or even cell-specific, local niches not representing at least 5% of the cells of at least one of the profiled sections were excluded from the analysis, as they did not represent general niches in the tissue, but rather unique rare microenvironments.

**Neighborhood Enrichment using Squidpy**

To characterize the spatial preference between cell types, we performed a neighborhood enrichment analysis using Squidpy^20^. In short, the neighborhood enrichment between cell type A and B represents the probability of assigning a cell-to-cell type A given the presence of a second cell of cell type B within a certain distance (d) divided by the probability of assigning a random cell to cell type A. Neighborhood enrichment scores were computed for each dataset and all the datasets collectively, identifying cell types localized in proximity across samples.

**Tumor Structure Scoring**

With the intention of quantitatively assessing the level of organization of the different profiled samples, we designed a metric named “Tissue Structure Score” (TSS). This metric employs the neighborhood enrichment scores obtained from paired cell types in each sample (see section above), resulting in a number that summarizes the level of organization of a profiled tissue.

The score is the sum of the absolute neighborhood enrichment scores. For each sample, we computed its Tumor Structure Score (TSS) as the mean of the absolute Z-score values obtained for all cell type pairs. The rationale behind this metric is that, in a completely disorganized tissue, no spatial preference will be found between any pair of cell types, keeping all neighborhood enrichment scores close to 0. On the contrary, very structured tissues will present extreme neighborhood enrichment scores, both positive and negative, resulting in a high TSS. This TSS score allowed us to classify samples depending on their organization into highly- (TSS > 4) and lowly-structured samples (TSS < 4). To validate this, we also checked the correlation between the TSS and the number of cells or the reads per cell. In both cases, the values were low (0.34 and 0.50, respectively), indicating that the TSS had a low correlation with the quality of the sample but saw a moderate correlation with the overall number of cells within the sample. An overview of the implementation can be seen in the GitHub repository (see Code Availability).

**TCGA Cohort Survival**

Clinical and genomic data for GB cases were obtained from The Cancer Genome Atlas (TCGA) using the TCGAbiolinks R package. To ensure consistency with the 2021 WHO Central Nervous System (CNS) Tumor Classification, we reclassified the TCGA diffuse glioma cohort according to Zakharova et al. 2022^28^. IDH-wildtype (IDHwt) glioblastomas were identified by filtering samples annotated as “Glioblastoma” with confirmed IDH wildtype status and included MGMT promoter status where available. Reclassified sample metadata were retrieved from Supplementary Table S2 of Zakharova et al. 2022. IDHwt GBM samples were subsets from the TCGA-GBM cohort based on case IDs matching reclassified annotations. The clinical data for these samples were merged with the reclassified metadata, ensuring the inclusion of vital status, survival times (days to last follow-up or death), age at diagnosis, sex, and MGMT promoter status. Cases missing key covariates were excluded to maintain data integrity for survival analysis.

Gene expression data for the TCGA-GBM cohort were queried from the Genomic Data Commons (GDC) portal. Transcriptomic profiling data generated via RNA-Seq (STAR - Counts workflow) were processed using DESeq2 to remove genes with fewer than ten counts across all samples. Normalized gene expression values were generated using variance stabilizing transformation (VST). Genes of interest associated with the Tumor Structured Score (TSS) were selected, and expression levels were aggregated into a module score by calculating the mean expression across genes. High and low TSS groups were defined based on the median module expression.

Survival outcomes were analyzed using the survival and survminer^30^ R packages. Overall survival (OS) was calculated as the time from diagnosis to death or last follow-up. Kaplan-Meier survival curves were generated for high and low TSS groups, and differences in survival distributions were assessed using the log-rank test. A multivariate Cox proportional hazards model was fitted to adjust for age, sex, and MGMT promoter status. The proportional hazards assumption was tested to validate the model. Survival plots with confidence intervals were generated using ggsurvplot, and p-values from log-rank tests were incorporated into the plots. Detailed code and datasets used in this study are available in a public GitHub repository (see Code Availability section).

**Microvasculature Assessment**

To explore the vasculature of the ISS samples, we first identified vessels. Vessels were defined as a group of endothelial and mural cells that are (1) tightly connected in space and (2) independent from other cells of their kind. By implementing and applying an algorithm that considers these conditions (see Code Availability), we define the number of vessels present in the profiled sections, their size, and their composition. To simplify the analysis of the datasets, we divided the vessels based on their size. In brief, we defined individual vessels detected based on the endothelial and mural cells detected within close proximity (20 microns) to each other, calculated spatial neighbors, and assigned each cell to a vessel. We then calculated the vessel size and classified them into groups: individual, small, large, and gigantic. Next, we calculated the composition of each vessel by size and assessed the genes associated with each vessel group. Next, we assessed the proportion of different cell types to the vessels and performed differential gene expression analysis based on the distance to the closest vessel for each cell type. Finally, we categorized each sample based on vessel presence and vessel size. An overview of the implementation can be seen in the GitHub repository(see Code Availability).

**Supplementary Tables**

**Supplementary Table 1:** Features of datasets included in the core and extended GBmap.

**Supplementary Table 2:** Gene signatures employed to perform automated cell typing using CellID.

**Supplementary Table 3:** Gene modules of each main cell territory obtained with HotSpot.

**Supplementary Table 4:** Differentially expressed genes for each annotation level (1 to 4) of the core GBmap.

**Supplementary Table 5:** Patient metadata of newly profiled GB by snRNA-seq and RNA-ISS.

**Supplementary Table 6:** Sequences of the padlock probes (PLPs) designed for RNA-ISS experiments.

**Supplementary Table 7:** Cox model results

**Methods references**

1 Wolf, F. A., Angerer, P. & Theis, F. J. SCANPY: large-scale single-cell gene expression data analysis. *Genome Biol* **19**, 15 (2018). <https://doi.org/10.1186/s13059-017-1382-0>

2 Kuemmerle, L. B. *et al.* Probe set selection for targeted spatial transcriptomics. *bioRxiv*, 2022.2008.2016.504115 (2022). <https://doi.org/10.1101/2022.08.16.504115>

3 Oh, S. *et al.* HGNChelper: identification and correction of invalid gene symbols for human and mouse. *F1000Res* **9**, 1493 (2020). <https://doi.org/10.12688/f1000research.28033.1>

4 McGinnis, C. S., Murrow, L. M. & Gartner, Z. J. DoubletFinder: Doublet Detection in Single-Cell RNA Sequencing Data Using Artificial Nearest Neighbors. *Cell Syst* **8**, 329-337 e324 (2019). <https://doi.org/10.1016/j.cels.2019.03.003>

5 Luecken, M. D. *et al.* Benchmarking atlas-level data integration in single-cell genomics. *Nature Methods* **19**, 41-50 (2022). <https://doi.org/10.1038/s41592-021-01336-8>

6 Clarke, Z. A. *et al.* Tutorial: guidelines for annotating single-cell transcriptomic maps using automated and manual methods. *Nat Protoc* **16**, 2749-2764 (2021). <https://doi.org/10.1038/s41596-021-00534-0>

7 Cortal, A., Martignetti, L., Six, E. & Rausell, A. Gene signature extraction and cell identity recognition at the single-cell level with Cell-ID. *Nature Biotechnology* **39**, 1095-1102 (2021). <https://doi.org/10.1038/s41587-021-00896-6>

8 Sun, D. *et al.* TISCH: a comprehensive web resource enabling interactive single-cell transcriptome visualization of tumor microenvironment. *Nucleic Acids Res* **49**, D1420-D1430 (2021). <https://doi.org/10.1093/nar/gkaa1020>

9 De Falco, A., Caruso, F., Su, X. D., Iavarone, A. & Ceccarelli, M. A variational algorithm to detect the clonal copy number substructure of tumors from scRNA-seq data. *Nat Commun* **14**, 1074 (2023). <https://doi.org/10.1038/s41467-023-36790-9>

10 Xu, C. *et al.* Probabilistic harmonization and annotation of single-cell transcriptomics data with deep generative models. *Molecular Systems Biology* **17**, e9620 (2021). <https://doi.org/https://doi.org/10.15252/msb.20209620>

11 Lotfollahi, M. *et al.* Mapping single-cell data to reference atlases by transfer learning. *Nature Biotechnology* (2021). <https://doi.org/10.1038/s41587-021-01001-7>

12 Traag, V. A., Waltman, L. & van Eck, N. J. From Louvain to Leiden: guaranteeing well-connected communities. *Sci Rep* **9**, 5233 (2019). <https://doi.org/10.1038/s41598-019-41695-z>

13 Becht, E. *et al.* Dimensionality reduction for visualizing single-cell data using UMAP. *Nature Biotechnology* **37**, 38-44 (2019). <https://doi.org/10.1038/nbt.4314>

14 Wu, H. *et al.* Mapping the single-cell transcriptomic response of murine diabetic kidney disease to therapies. *Cell Metab* **34**, 1064-1078 e1066 (2022). <https://doi.org/10.1016/j.cmet.2022.05.010>

15 DeTomaso, D. & Yosef, N. Hotspot identifies informative gene modules across modalities of single-cell genomics. *Cell Systems* **12**, 446-456.e449 (2021). <https://doi.org/https://doi.org/10.1016/j.cels.2021.04.005>

16 Lopez, R., Regier, J., Cole, M. B., Jordan, M. I. & Yosef, N. Deep generative modeling for single-cell transcriptomics. *Nat Methods* **15**, 1053-1058 (2018). <https://doi.org/10.1038/s41592-018-0229-2>

17 Hao, Y. *et al.* Integrated analysis of multimodal single-cell data. *Cell* **184**, 3573-3587.e3529 (2021). <https://doi.org/https://doi.org/10.1016/j.cell.2021.04.048>

18 inferCNV of the Trinity CTAT Project (2019).

19 Qian, X. *et al.* Probabilistic cell typing enables fine mapping of closely related cell types in situ. *Nature Methods* **17**, 101-106 (2020). <https://doi.org/10.1038/s41592-019-0631-4>

20 Palla, G. *et al.* Squidpy: a scalable framework for spatial omics analysis. *Nat Methods* **19**, 171-178 (2022). <https://doi.org/10.1038/s41592-021-01358-2>

21 Ravi, V. M. *et al.* Spatially resolved multi-omics deciphers bidirectional tumor-host interdependence in glioblastoma. *Cancer Cell* **40**, 639-655.e613 (2022). <https://doi.org/10.1016/j.ccell.2022.05.009>

22 Kleshchevnikov, V. *et al.* Cell2location maps fine-grained cell types in spatial transcriptomics. *Nat Biotechnol* (2022). <https://doi.org/10.1038/s41587-021-01139-4>

23 Jin, S. *et al.* Inference and analysis of cell-cell communication using CellChat. *Nature Communications* **12**, 1088 (2021). <https://doi.org/10.1038/s41467-021-21246-9>

24 Ramilowski, J. A. *et al.* A draft network of ligand-receptor-mediated multicellular signalling in human. *Nat Commun* **6**, 7866 (2015). <https://doi.org/10.1038/ncomms8866>

25 Li, Z., Wang, T., Liu, P. & Huang, Y. SpatialDM for rapid identification of spatially co-expressed ligand-receptor and revealing cell-cell communication patterns. *Nat Commun* **14**, 3995 (2023). <https://doi.org/10.1038/s41467-023-39608-w>

26 Cang, Z. *et al.* Screening cell-cell communication in spatial transcriptomics via collective optimal transport. *Nat Methods* **20**, 218-228 (2023). <https://doi.org/10.1038/s41592-022-01728-4>

27 Kukanja, P. et al. Cellular architecture of evolving neuroinflammatory lesions and multiple sclerosis pathology. Cell 187, 1990-2009.e19 (2024). <https://doi.org/10.1016/j.cell.2024.02.030>.

28 Zakharova, G. et al. Reclassification of TCGA Diffuse Glioma Profiles linked to transcriptomic, epigenetic, genomic and clinical data, according to the 2021 WHO CNS tumor classification. *Int J Mol Sci* **24**, 157 (2022). <https://doi.org/10.3390/ijms24010157>.

29 Weigert, M. & Schmidt, U. Nuclei instance segmentation and classification in histopathology images with Stardist. IEEE Int Symp Biomed Imaging Challenges (ISBIC) (2022). <https://doi.org/10.1109/ISBIC56247.2022.9854534>.

30 Kassambara, A., Kosinski, M. & Biecek, P. *survminer: Drawing Survival Curves using 'ggplot2'*. R package version 0.5.0 (2024). [https://CRAN.R-project.org/package=survminer](https://cran.r-project.org/package=survminer).
